# Shape-shifting trypanosomes: Flagellar shortening followed by asymmetric division in *Trypanosoma congolense* from the tsetse proventriculus

**DOI:** 10.1371/journal.ppat.1007043

**Published:** 2018-05-17

**Authors:** Lori Peacock, Christopher Kay, Mick Bailey, Wendy Gibson

**Affiliations:** 1 School of Biological Sciences, University of Bristol, Bristol, United Kingdom; 2 Bristol Veterinary School, University of Bristol, Langford, Bristol, United Kingdom; University of Texas Medical School at Houston, UNITED STATES

## Abstract

Trypanosomatids such as *Leishmania* and *Trypanosoma* are digenetic, single-celled, parasitic flagellates that undergo complex life cycles involving morphological and metabolic changes to fit them for survival in different environments within their mammalian and insect hosts. According to current consensus, asymmetric division enables trypanosomatids to achieve the major morphological rearrangements associated with transition between developmental stages. Contrary to this view, here we show that the African trypanosome *Trypanosoma congolense*, an important livestock pathogen, undergoes extensive cell remodelling, involving shortening of the cell body and flagellum, during its transition from free-swimming proventricular forms to attached epimastigotes *in vitro*. Shortening of the flagellum was associated with accumulation of PFR1, a major constituent of the paraflagellar rod, in the mid-region of the flagellum where it was attached to the substrate. However, the PFR1 depot was not essential for attachment, as it accumulated several hours after initial attachment of proventricular trypanosomes. Detergent and CaCl_2_ treatment failed to dislodge attached parasites, demonstrating the robust nature of flagellar attachment to the substrate; the PFR1 depot was also unaffected by these treatments. Division of the remodelled proventricular trypanosome was asymmetric, producing a small daughter cell. Each mother cell went on to produce at least one more daughter cell, while the daughter trypanosomes also proliferated, eventually resulting in a dense culture of epimastigotes. Here, by observing the synchronous development of the homogeneous population of trypanosomes in the tsetse proventriculus, we have been able to examine the transition from proventricular forms to attached epimastigotes in detail in *T*. *congolense*. This transition is difficult to observe *in vivo* as it happens inside the mouthparts of the tsetse fly. In *T*. *brucei*, this transition is achieved by asymmetric division of long trypomastigotes in the proventriculus, yielding short epimastigotes, which go on to colonise the salivary glands. Thus, despite their close evolutionary relationship and shared developmental route within the vector, *T*. *brucei* and *T*. *congolense* have evolved different ways of accomplishing the same developmental transition from proventricular form to attached epimastigote.

## Author summary

Tsetse-transmitted trypanosomes are parasitic protists that cause severe human and livestock diseases in tropical Africa. During their developmental cycle in the tsetse fly, these trypanosomes undergo complex cycles of differentiation and proliferation. Here we have investigated part of the developmental cycle of the major livestock pathogen *Trypanosoma congolense* as it moves from the fly midgut via the foregut to the mouthparts, where it reacquires infectivity to mammalian hosts. This transition is difficult to observe *in vivo* because of the small numbers of migratory trypanosomes and their inaccessibility in the fly. However, prior to migration, trypanosomes accumulate in the proventriculus, the valve that separates the foregut from the midgut, and we were able to observe the behaviour of these cells *in vitro*. On release from the proventriculus, these trypanosomes readily attach to a glass microscope slide and then undergo drastic remodelling to become short, stout cells, before each produces a small daughter cell. Each mother cell goes on to produce at least one further daughter trypanosome in the same way, while the daughter cells also proliferate as attached cells. We assume that these events would normally happen *in vivo* inside the tsetse proboscis. In *T*. *brucei* the equivalent developmental transition takes place in the proventriculus or foregut in free-swimming rather than attached cells, and is achieved via an asymmetric division. Thus, despite their close evolutionary relationship, these two trypanosome species have evolved different ways of accomplishing what is essentially the same developmental transition.

## Introduction

**Fig 1:**
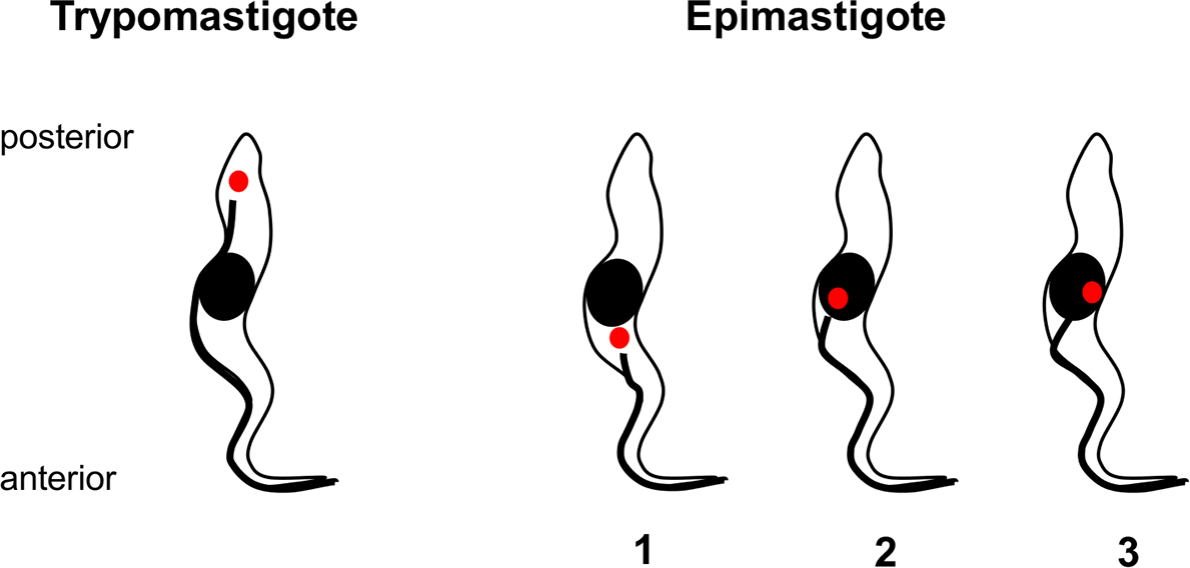
Diagram comparing trypomastigote and epimastigote morphology. Black oval represents the nucleus; small red circle represents the kinetoplast, an organelle containing the tightly packaged mitochondrial DNA. In trypomastigotes (left) the kinetoplast is posterior to the nucleus, whereas in epimastigotes (right) the kinetoplast is typically anterior to the nucleus (1). In this category, though not as classically defined, we also include trypanosomes with the kinetoplast juxtaposed to the nucleus as shown in examples 2 and 3. The flagellum (thick black line) arises from a basal body that is physically linked to the kinetoplast [37].

Trypanosomatids such as *Leishmania* and *Trypanosoma* are digenetic, single-celled, parasitic flagellates that undergo complex life cycles involving morphological and metabolic changes to fit them for survival in different environments within their hosts. While metabolic changes are brought about by changes in gene expression, a consensus has emerged from recent studies that gross morphological transitions are accomplished by asymmetric division rather than cell remodelling. For example, in *Leishmania* and *Trypanosoma cruzi* the invasion of mammalian cells involves drastic shortening or loss of the flagellum, which is achieved by asymmetric division to produce an amastigote daughter cell from a progenitor with a long flagellum [1,2]. In the African trypanosomes, *T*. *brucei* and *T*. *vivax*, asymmetric division enables the rearrangement of cellular organelles (Fig 1) necessary for the trypomastigote-epimastigote transitions that are an intrinsic part of the developmental cycle in tsetse [3–5]. Here we focus on the trypomastigote-epimastigote transition in the related African tsetse-transmitted trypanosome, *T*. *congolense* savannah.

In tropical Africa tsetse-transmitted trypanosomes such as *T*. *brucei*, *T*. *congolense* and *T*. *vivax* are severely detrimental to livestock health and productivity. These trypanosomes undergo a complex life cycle in the tsetse fly vector, starting with ingestion of blood from an infected host and culminating in the production of mammal infective stages (metacyclics) coated with variant surface glycoprotein. In *T*. *congolense* savannah, trypanosomes in the blood meal differentiate into procyclic stages in the fly midgut and then rapidly multiply before invading the proventriculus (or cardia), the valve between the foregut and midgut. Within the proventriculus, trypanosomes accumulate in a dense, non-dividing population of elongated trypomastigotes [6] and then migrate forwards to invade the mouthparts. Here they attach to the walls of the food canal and cibarial pump [7–11] and become epimastigotes, the morphological form where the kinetoplast is anterior to the nucleus (Fig 1). These forms proliferate and subsequently invade the hypopharynx, the narrow tube that conveys saliva from the salivary glands to the tip of the proboscis. Trypanosomes continue to proliferate in the hypopharynx, and both attached and free trypanosomes are found within its lumen. Trypanosomes revert to trypomastigote morphology (kinetoplast posterior to the nucleus, Fig 1), and finally metacyclics are produced, in as little as 19 days after the infected blood meal [12].

The low numbers of trypanosomes present within an individual fly and their restricted location make it difficult to study the initial steps of invasion and colonisation of the mouthparts, but the whole life cycle of *T*. *congolense* savannah can be recapitulated *in vitro* [13], including growth of epimastigotes attached to glass or plastic surfaces [14]. Epimastigote cultures have been established from primary cultures of infected tsetse organs (proboscis, foregut or midgut) grown with explants of bovine dermal collagen; here, the presence of proventricular trypanosomes was considered to be the key to success [15]. Colonies of attached epimastigotes also arise spontaneously in long term cultures of procyclics [13], but it is unclear what triggers the transition. Under natural conditions, the transition occurs when proventricular trypomastigotes reach the fly mouthparts and attach to the walls of the food canal [12]. Before migration, these trypanosomes constitute a homogeneous population of non-dividing cells at high density within the tsetse proventriculus [6], making it possible to isolate them from dissected proventriculi and study their development *in vitro*. Here we show that the trypomastigote-epimastigote transition is accomplished in these stages: attachment of trypomastigotes to the substrate via the flagellum, shortening of the cell body and flagellum, followed by an asymmetric division yielding a small epimastigote. Shortening of the mature flagellum is without precedent in trypanosomes [16], and here was accompanied by the accumulation of a major constituent protein of the paraflagellar rod, PFR1, in the mid-region of the flagellum.

## Results

### Remodelling of proventricular trypanosomes

**Fig 2:**
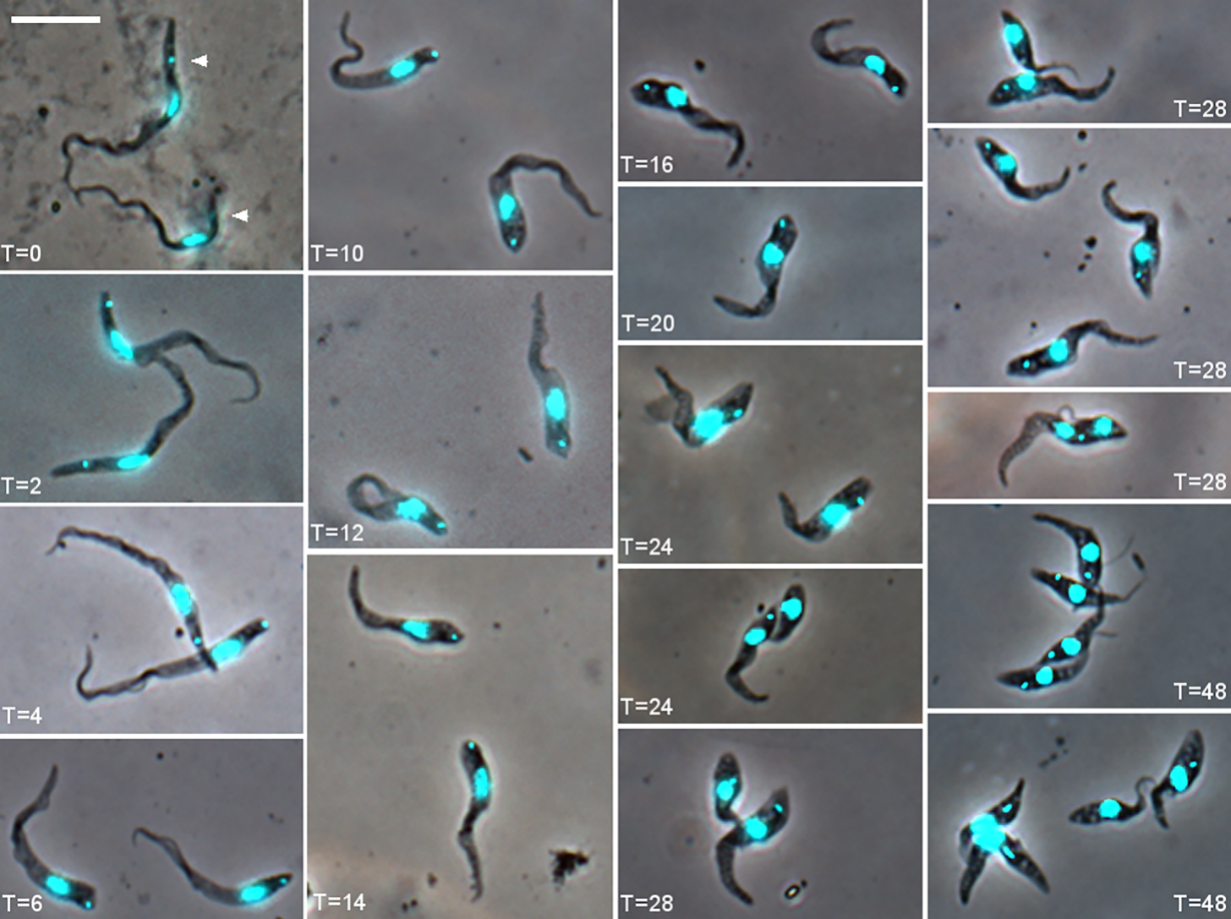
Time course of development of *Trypanosoma congolense* 1/148 YPFR *in vitro*. Trypanosomes were imaged from time zero (T = 0) to 48 hours (T = 48) after seeding cultures from pooled proventricular forms. Each image, except T = 0, represents trypanosomes attached to the coverslip, as free-swimming cells were washed away. Brightfield image merged with the DAPI image (recoloured cyan for clarity). The kinetoplast is indicated by an arrowhead in each trypanosome at T = 0 and is easy to identify in all trypanosomes shown up to T = 20; in the dividing trypanosomes at T = 24 to T = 48, the kinetoplast is often juxtanuclear in the daughter cell and therefore indistinct. Scale bar = 10 μm.

Proventriculi dissected from infected tsetse were pooled, releasing proventricular trypanosomes, which were then incubated in culture medium. Within 30 minutes of transfer to culture, most proventricular trypanosomes had attached to the glass coverslip by their anterior end (S1 Movie). While some trypanosomes formed a permanent attachment, others attached temporarily before moving on to attach elsewhere (S2 Movie). Over the next few hours, the cell shape was extensively remodelled becoming progressively shorter and stouter, but retaining the trypomastigote conformation (i.e. with the kinetoplast posterior to the nucleus) (Fig 2; S1 Movie). The point of attachment of the flagellum to the substrate also changed over time; initial attachment was via the anterior tip of the flagellum, but the attachment zone subsequently moved to the mid-region of the flagellum, such that the anterior tip of the trypanosome was able to resume movement (S2 Movie). Cells then entered cell division (S3 Movie) and after two days the population consisted of small clusters of two to four trypanosomes, which were a mixture of trypo- and epimastigotes with the kinetoplast closely adjacent or anterior to the nucleus (Fig 1). This pattern of development–attachment, shortening, cell division, formation of clusters of attached cells—was seen in proventricular trypanosomes of four different strains of *T*. *congolense* savannah (1/148, WG81, Gam 2 and S104), demonstrating that it is a programmed developmental pathway.

Image and morphometric data were collected from overlapping sequential time courses from T = 0 to T = 60 hours for strain 1/148 YPFR. The nucleus and kinetoplast were stained with DAPI, while the flagellum was visualised by fluorescently tagging one of the major structural proteins of the paraflagellar rod (PFR1 [17,18]); the PFR extends almost the whole length of the flagellum, starting where the flagellum exits the flagellar pocket and running parallel to the axoneme but does not extend into the free flagellum [7,19]. Representative cells stained with DAPI are shown in Fig 2, commencing at T = 0 with long proventricular trypomastigotes from the fly. From T = 2 onwards, the cell shape changed profoundly: the overall cell length decreased and the posterior became blunt and rounded rather than narrow and pointed (compare Fig 2 T = 2 and T = 10); the width of the trypanosome increased and the nucleus, at first sausage-shaped, became rounder. Cells had a single kinetoplast and nucleus (1K1N) until T = 14, when 2K1N cells started to appear in the population; some cells had already completed cell division by T = 24 producing a smaller daughter cell, and by T = 48, there were clusters of actively dividing trypo- and epimastigotes (Fig 2).

Mensural data for trypanosomes T = 0 to T = 14 before the onset of first cell division is compiled in S1 Table; S1 Fig shows the measurements made on each trypanosome. Variables are plotted individually against time in S2 Fig.

### Shortening of the flagellum

**Fig 3:**
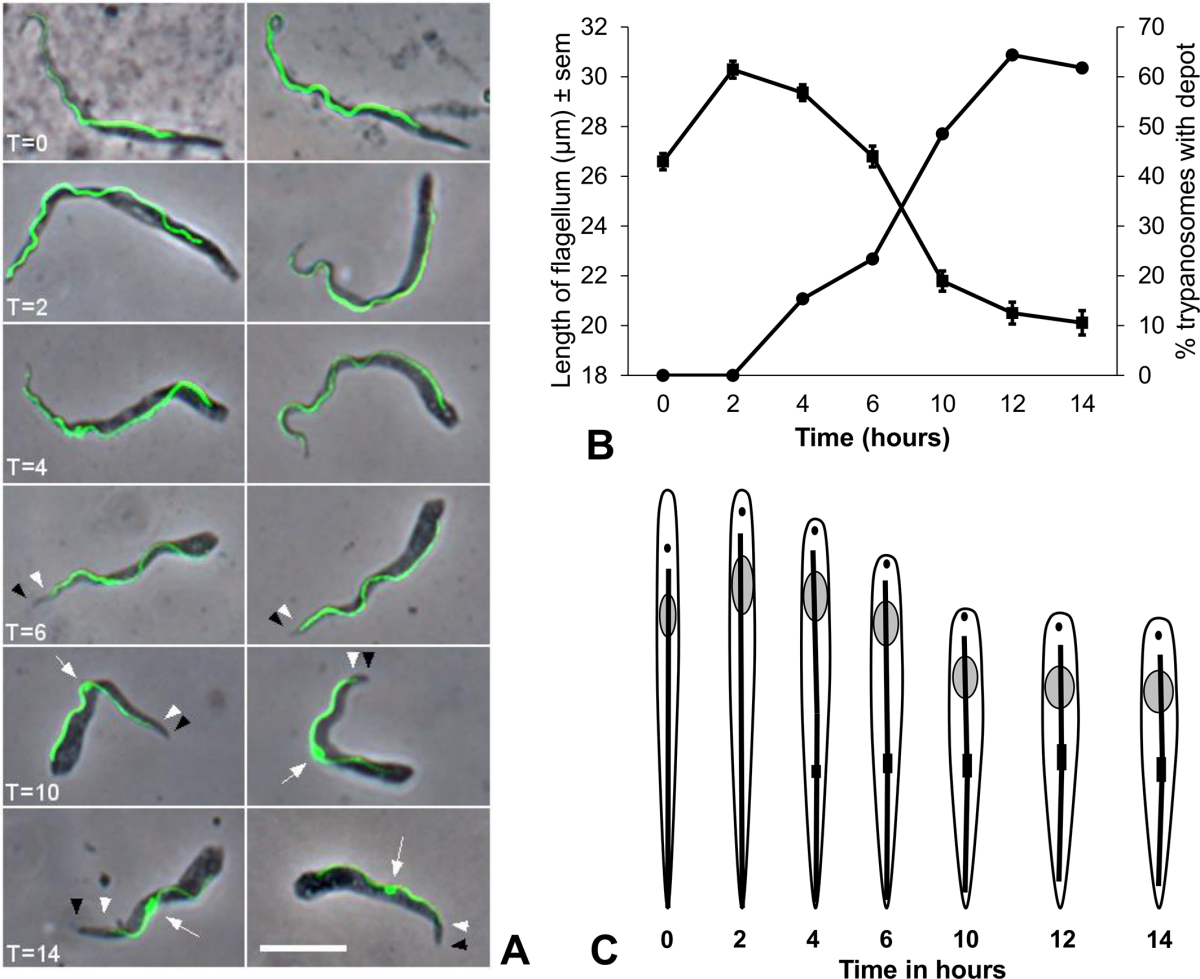
Shortening of flagellum. **(A)** Images from *Trypanosoma congolense* 1/148 YPFR time course showing decrease of flagellar length (visualised by YFP::PFR1 fluorescence) over time; details as Fig 2 legend. Note first appearance of PFR1 depot at T = 10 (arrows). The arrowheads indicate that the PFR does not extend to end of cell body (T = 6 to T = 14); white arrowhead, end of PFR; black arrowhead, end of cell body. Brightfield image merged with the YFP image (YFP::PFR1). Scale bar = 10 μm. **(B)** Length of flagellum (squares) and percentage of trypanosomes with YFP::PFR1 depot (circles) from T = 0 to T = 14. **(C)** Diagrammatic representation of morphological changes in trypanosomes from T = 0 to T = 14 compiled from morphometric data (S1 Table, S3 Table). The PFR is indicated by the thick black line, with depot shown as a filled rectangle.

As part of the remodelling process, after an initial increase, the flagellum decreased by approximately 30% in length—from 30 to 20 μm as judged by PFR length in YFP::PFR1 trypanosomes—and it was evident that the PFR no longer extended as far as the anterior end of the cell body (Fig 3). The shortening of the flagellum was accompanied by a noticeable accumulation of YFP::PFR1 in the mid-region of the cell (Fig 3A). Cells with a depot of YFP::PFR1 started to appear at T = 4 hours, and the proportion increased to approximately 65% during the first 14 hours of the time course, coincident with decrease in flagellar length (Fig 3B and 3C). The PFR1 depot is not an artifact of fixation, because it was clearly visible in live cells (S4 Movie).

**Fig 4:**
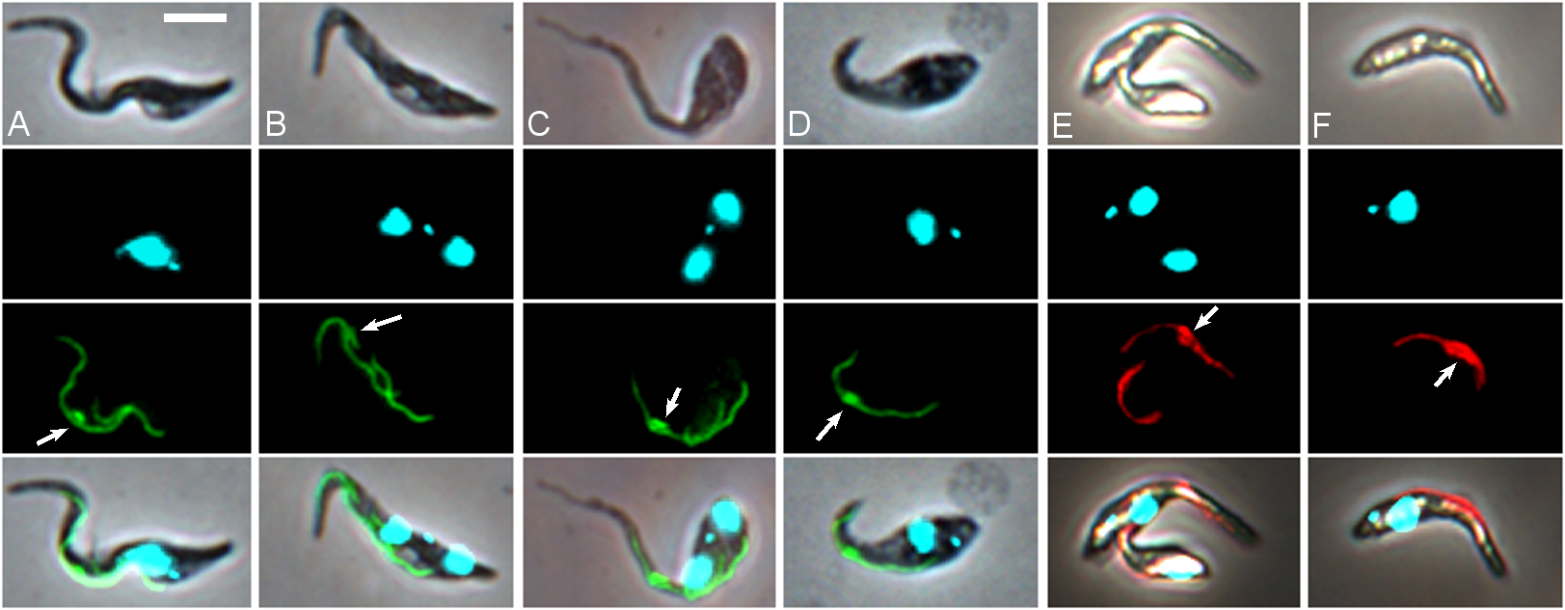
Accumulation of PFR1. Proventricular trypanosomes from *in vitro* culture showing accumulation of YFP::PFR1 depot (arrows) in *T*. *congolense* WG81 YPFR (A, B) and S104 YPFR (C, D), and of RFP::PFR1 depot (arrows) in WG81 RPFR (E, F). Rows top to bottom: brightfield, DAPI, YFP or RFP, merge. Scale bar = 5 μm.

It became harder to identify depot unequivocally in dividing cells, as the intensity of YFP fluorescence is greater in regions where the old and new flagella overlap. Nevertheless, it was observed that depot often persisted in the mother cell through the first cell division, alongside the growth of a new flagellum in the daughter cell (Table 1, S4 Movie), indicating that YFP::PFR1 building blocks were not being recycled for construction of the new flagellum. The accumulation of YFP::PFR1 was a consistent feature of remodelling proventricular cells in all three trypanosome strains examined (1/148, WG81, S104; Figs 3A and 4), and also when mRFP was substituted for YFP as the fluorescent protein (WG81 RPFR, Fig 4), demonstrating accumulation of PFR1 using two different protein tags.

**Table 1:** Presence of a depot of YFP::PFR1 in *T*. *congolense* 1/148 proventricular forms during the first cell division. A total of 355 pairs of dividing cells were scored. Mother and daughter cells were distinguished by their relative positions and cell shape.

Cell identity	Depot of YFP::PFR1	
	Observed	Not observed
Mother cell	220/355 (62.0%)	135/355 (38.0%)
Daughter cell	6/355 (1.7%)	349/355 (98.3%)

We noticed that there appeared to be no difference in the intensity of fluorescence of the PFR of the mother and daughter cells, in contrast to our previous observations of *T*. *brucei* transfected with the same construct, where the paler daughter flagellum was easily distinguished from the old one [20]. S3 Fig compares YFP::PFR1 *T*. *brucei* and *T*. *congolense* procyclics in various stages of division, confirming that there is no visible difference in fluorescence between the old and new flagella of *T*. *congolense* in contrast to *T*. *brucei*.

### Flagellar attachment

**Fig 5:**
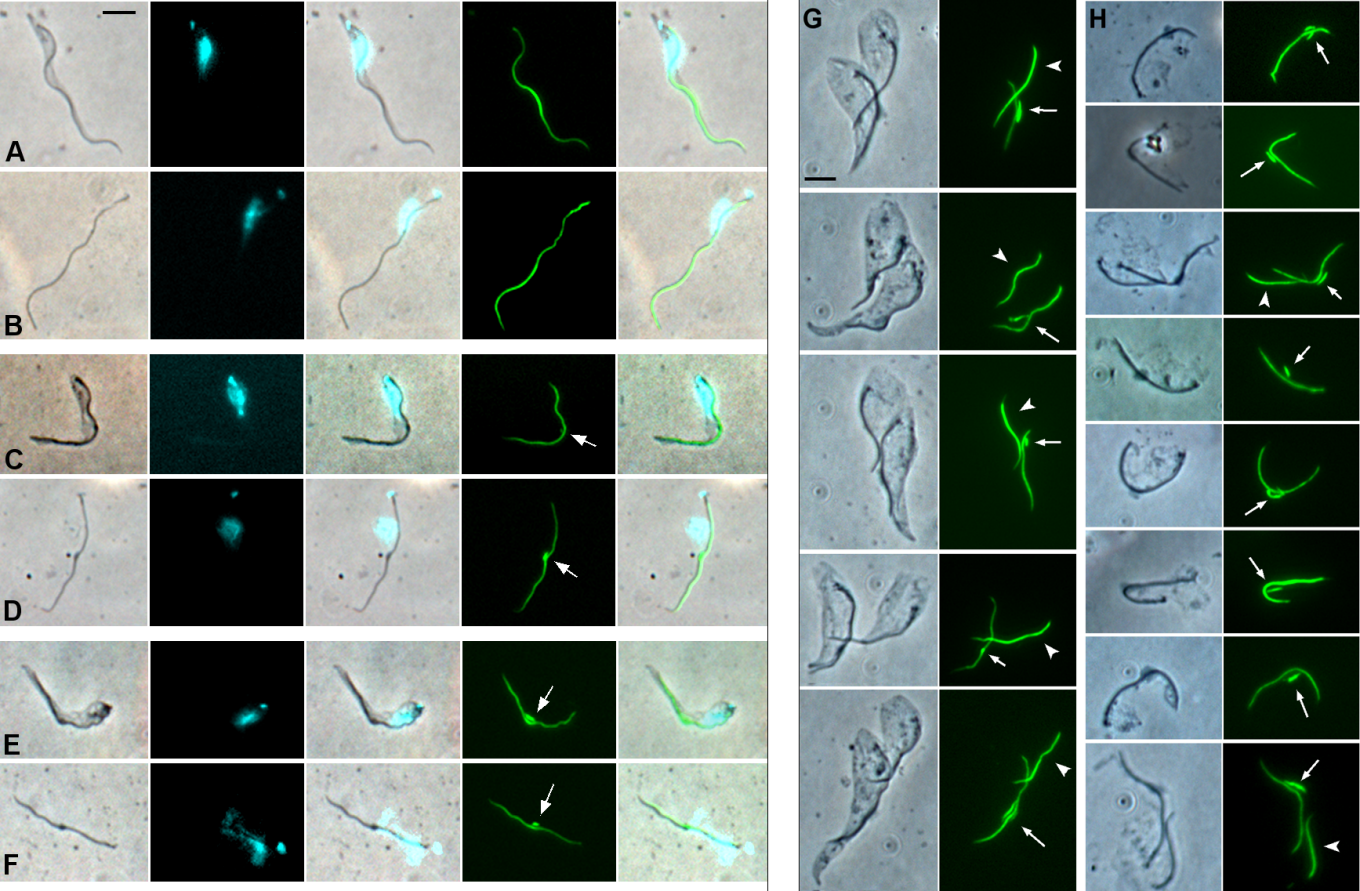
Analysis of flagellar attachment in cytoskeleton preparations. Cytoskeletons of *Trypanosoma congolense* 1/148 YPFR were prepared using 0.5% Triton (rows A, C, E and column G) or 0.5% Triton followed by CaCl_2_ treatment to selectively remove subpellicular microtubules (rows B, D, F and column H) [21]. Rows A and B after 1 hour incubation; rows C and D after 6 hours; rows E and F after 12 hours and columns G and H after 24 hours. Arrows indicate YFP::PFR1 depot; arrowheads indicate PFR of daughter flagellum (columns G and H). Rows A–F, L to R: brightfield, DAPI, merge, YFP, merge. The DAPI staining is more dispersed than usual, because of membrane disruption by detergent. Columns G and H, L to R: brightfield, YFP. Scale bar = 5 μm.

To investigate the nature of attachment of the flagellum to the glass coverslip, we treated proventricular cells with the detergent Triton X-100 to remove the cell membrane leaving the flagellum and subpellicular microtubule cytoskeleton intact [21]. We found that detergent treatment did not disrupt attachment of proventricular trypanosomes after 1 hour, 6 hours, 12 hours or 24 hours in culture (Fig 5A, 5C, 5E and 5G), as previously shown for epimastigotes in continuous culture [21] and confirmed here (S4 Fig). Additional treatment with calcium chloride to disrupt subpellicular microtubules [21] left the flagella attached to the glass substrate (Fig 5B, 5D, 5F and 5H), demonstrating that attachment is not dependent on membrane binding to substrate but is via the flagellum. The accumulation of PFR1 occurred independent of attachment, as it was not present in attached cytoskeletons after 1 hour in culture (Fig 5A and 5B) and appeared at later stages of incubation (6 and 12 hours, Fig 5C–5F). In dividing cells after 24 hours in culture, both mother and daughter cytoskeletons were present after detergent treatment (Fig 5G), but most of the daughter cells disappeared after calcium chloride treatment (Fig 5H), showing that they are not yet attached to substrate by the flagellum and are presumably connected to the mother cell by interactions of the subpellicular microtubules.

### Analysis of morphometric data

**Fig 6:**
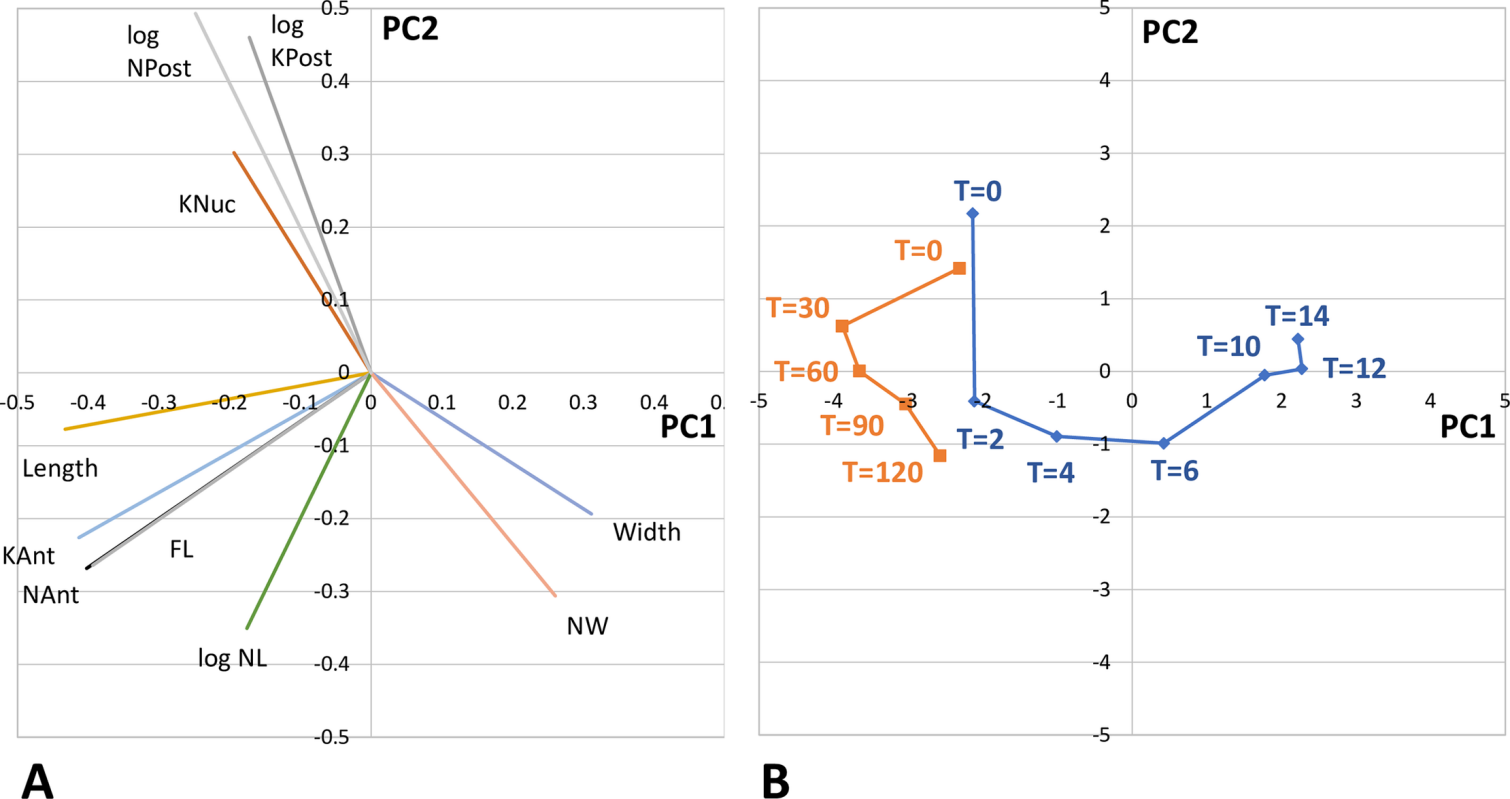
Principal component analysis of cell morphometrics. Data from *Trypanosoma congolense* 1/148 YPFR time course as Fig 2 legend. (**A**) PC1 and PC2 loading vectors for ten variables; all variables conformed to a normal distribution except for NPost, KPost and NL, which were therefore log transformed. (**B**) Mean values of PC1 and PC2 for trypanosome population at each time point. Data set T = 0 to T = 14 hours in blue; data set T = 0 to T = 120 minutes in orange. Data sets are in S1 Table and S2 Table.

To investigate the morphological changes involved in remodelling proventricular cells before the visible onset of cell division, we analysed ten morphometric variables for T = 0 to T = 14 (S1 Table) by principal component analysis (PCA). PCA groups variables that co-vary and allows the detection of underlying latent variables (Fig 6). Two latent variables, PC1 and PC2, described most of the morphological variation (50.3% and 20.8% respectively). Four variables contributed most to PC1: there was a negative association with NAnt and KAnt (distances from the cell anterior to the nucleus and kinetoplast respectively–see S1 Fig), and with length and FL (flagellar length) (Fig 6A). Two variables showed a strong positive association with PC2: NPost and KPost (distances from the cell posterior to the nucleus and kinetoplast respectively; Fig 6A). Loadings for PC1 and PC2 are given in S4 Table.

When PC2 was plotted against PC1, rather than a smooth transition from the long proventricular form to the short, stout trypomastigote, distinct phases of remodelling were evident in the 14 hour time course (Fig 6B, blue): during the first two hours there was a sharp decrease in PC2, followed by an increase in PC1 from T = 2 to T = 6 hours, then a phase when both PC1 and PC2 increased, with a final abrupt increase in PC2 from T = 12 to T = 14 hours. Data points for individual trypanosomes at each time point are presented as an animated GIF (S1 File). In summary, there are discrete phases of cellular remodelling as the trypanosomes progress along their developmental pathway from non-dividing proventricular cells to initiation of cell division at T = 14 hours.

Fig 3C summarises the morphological changes over time T = 0 to T = 14. Paradoxically, remodelling during the first two hours involved an increase in flagellar length, although cell length remained constant (S2 Fig). To investigate this anomaly, we carried out a finer time course, with observations every 30 minutes over the first two hours on attached cells (S2 Table and S5 Fig). The initial increase in flagellum length occurred during the first 30 minutes, together with an increase in cell length, which was not picked up in the 14 hour time course, because it is transient (S5 Fig). This is reflected in the PCA (Fig 6B, orange), where there is the same decline in PC2 seen in the 14 hour time course (Fig 6B, blue), but now accompanied by a sharp decrease in PC1 during the first 30 minutes. This time period correlates with attachment of proventricular trypanosomes to the coverslip, which occurred within the first 30 minutes for the majority of cells. Considering that this initial increase in length is very fast and runs counter to the trend thereafter for the cell to shorten, we think that attachment causes a conformational change in trypanosome shape as the cell flattens onto the glass surface and becomes constrained in 2D. During the first two hours, there was also a coordinated decrease in NPost and KPost (distances from the cell posterior to the nucleus and kinetoplast respectively), and a corresponding increase in NAnt and KAnt (distances from the cell anterior to the nucleus and kinetoplast respectively), while cell length and KNuc, the distance between the nucleus and kinetoplast, remained constant (S1 Table, Fig 3C and S5 Fig). This indicates movement of the nucleus and kinetoplast towards the posterior rather than shortening of the posterior end.

From T = 2 to T = 10 hours, there was a major decrease in cell and flagellar length, together with NAnt and KAnt (distances from the cell anterior to the nucleus and kinetoplast respectively; S2 Fig). The shape of a trypanosome is defined by a corset of subpellicular microtubules, with their plus ends at the posterior of the cell [19], so decrease in cell length would be achieved either by removal of tubulin subunits from individual microtubules, or slippage such that the microtubules forming the pointed posterior tip move anteriorly between their fellows. The fact that cell width also increased markedly (S2 Fig) suggests the latter mechanism, allowing the trypanosomes to become both shorter and fatter, but it is also possible that new microtubules were assembled between the existing ones. From T = 10 to T = 14 hours, the rate of change in cell and flagellar length flattens off (S2 Fig), indicating that the major remodelling of the cell is now complete and it is ready to initiate cell division.

### First cell division

**Fig 7:**
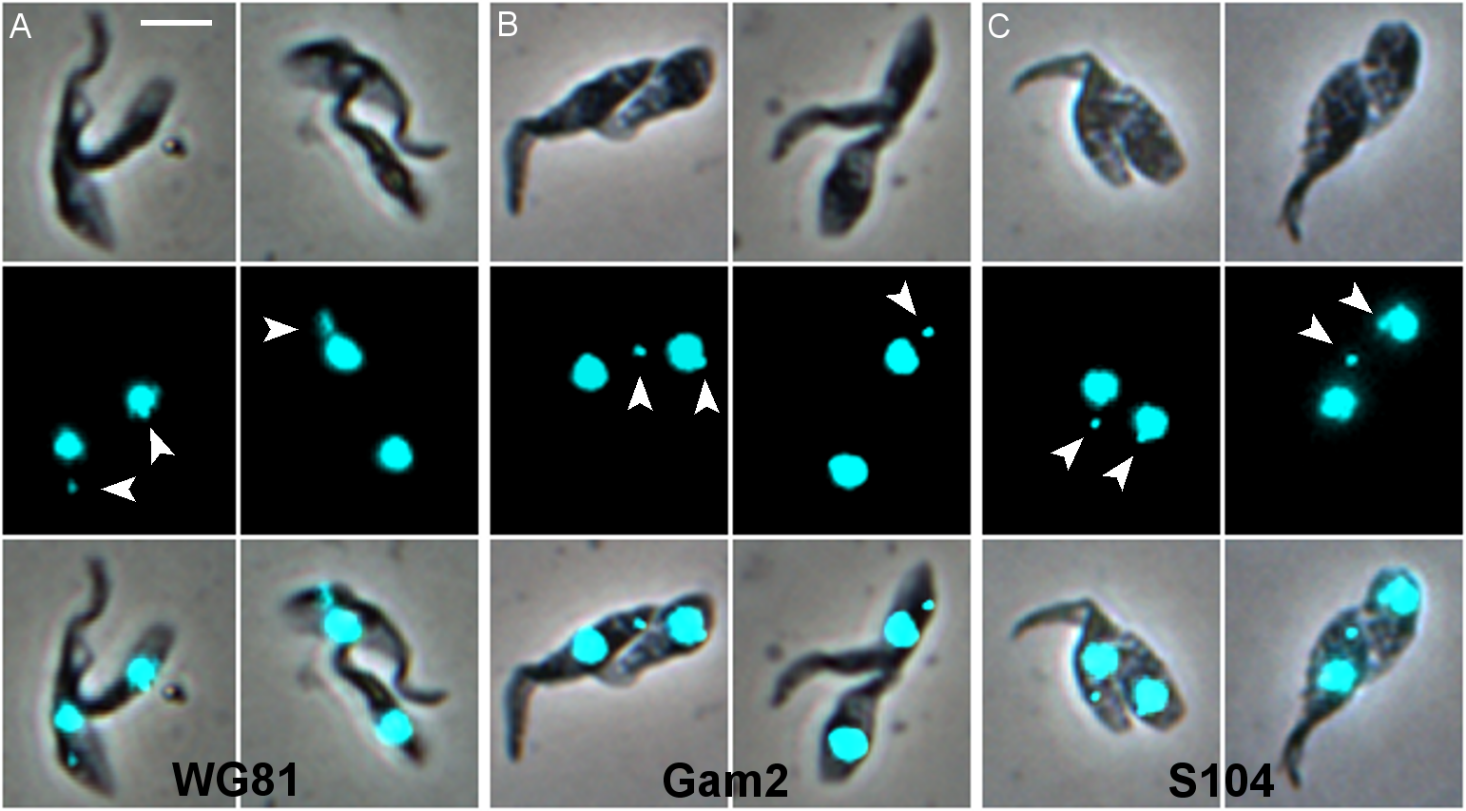
First cell division of proventricular forms of *T*. *congolense*. Cultured proventricular forms from three different strains of *T*. *congolense*: WG81 (A), Gam2 (B), S104 (C). Rows top to bottom: brightfield, DAPI, merge. The white arrowheads indicate the kinetoplast; where the kinetoplast of the daughter cell is not indicated, it is presumably juxtanuclear and therefore hidden (columns 2 and 4). In column 2, the kinetoplast of the mother cell is elongated, indicating that a further round of cell replication has begun. Scale bar = 5 μm.

The first cell division resulted in a small trypanosome (Fig 2), apparently tethered to the centre of the mother cell by its relatively long free flagellum (S3, S4 and S5 Movies). This unusual division was observed in proventricular forms for three other strains of *T*. *congolense* besides 1/148 (Fig 7). It is not an artifact of *in vitro* culture, because the same morphology was observed in dividing cells from the proboscis (S6 Movie). We did not observe these cells in our previous study [6], probably because we examined established proboscis infections rather than the early stage of colonization by small numbers of proventricular trypanosomes. Moreover, as these cells are firmly attached to the substrate, we would not have found them without disrupting the proboscis.

**Fig 8:**
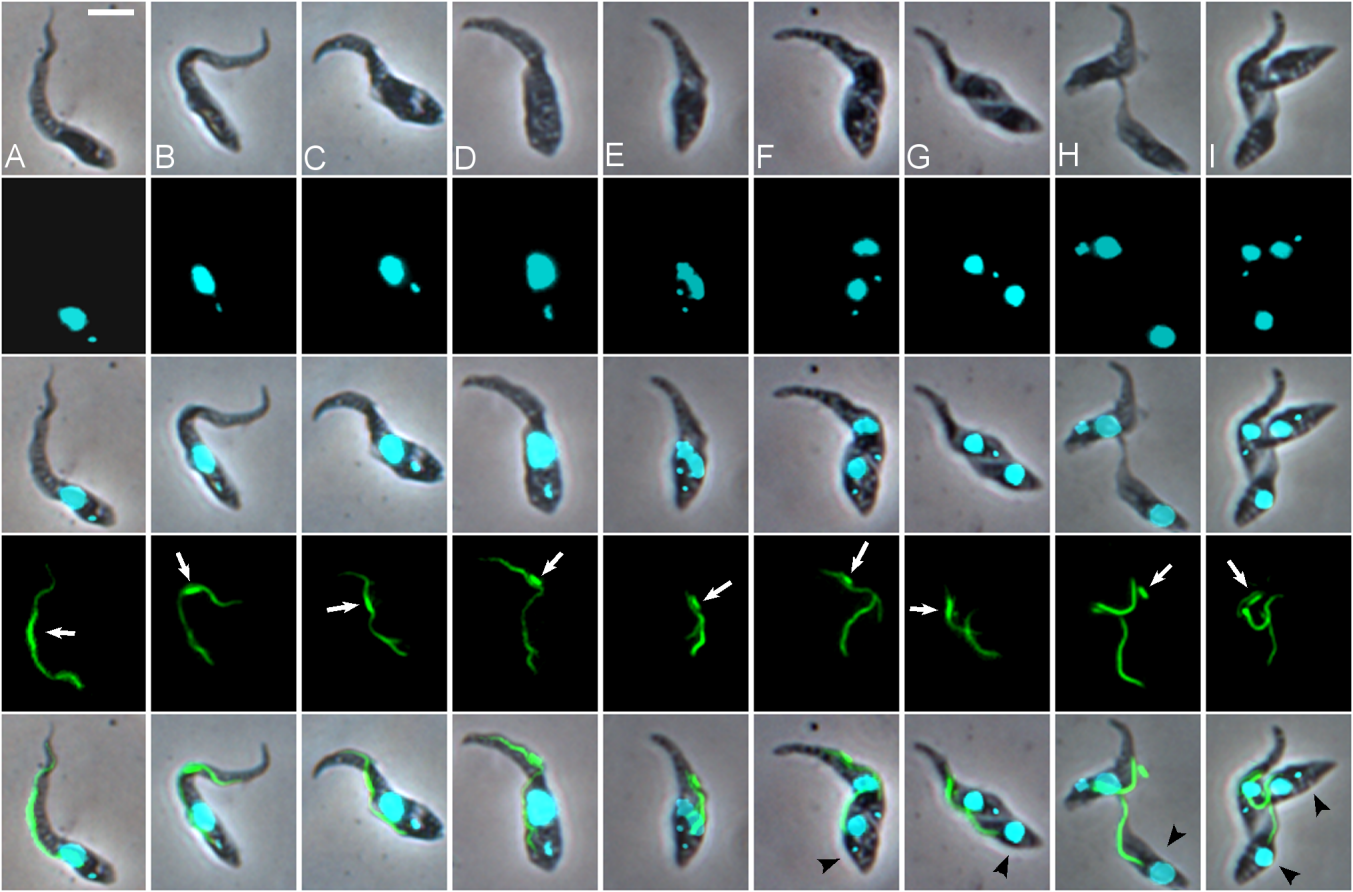
Sequence of events in formation of first and second daughter cells. Cultured proventricular forms of *T*. *congolense* 1/148 YPFR. **(A)** Cell prior to division, 1K1N; **(B)** elongated kinetoplast, 1K1N; **(C, D)** enlarged kinetoplast and growth of new flagellum, 1K1N; **(E)** nuclear division, 2K1N; **(F, G)** formation of cleavage furrow, 2K2N; **(H)** mother cell with attached daughter; enlarged kinetoplast and new flagellum indicate that mother cell is dividing again; **(I)** rosette of three cells. White arrows indicate YFP::PFR1 depot; black arrowheads indicate daughter cells. Rows top to bottom: brightfield, DAPI, merge, YFP, merge. Scale bar = 5 μm.

**Fig 9:**
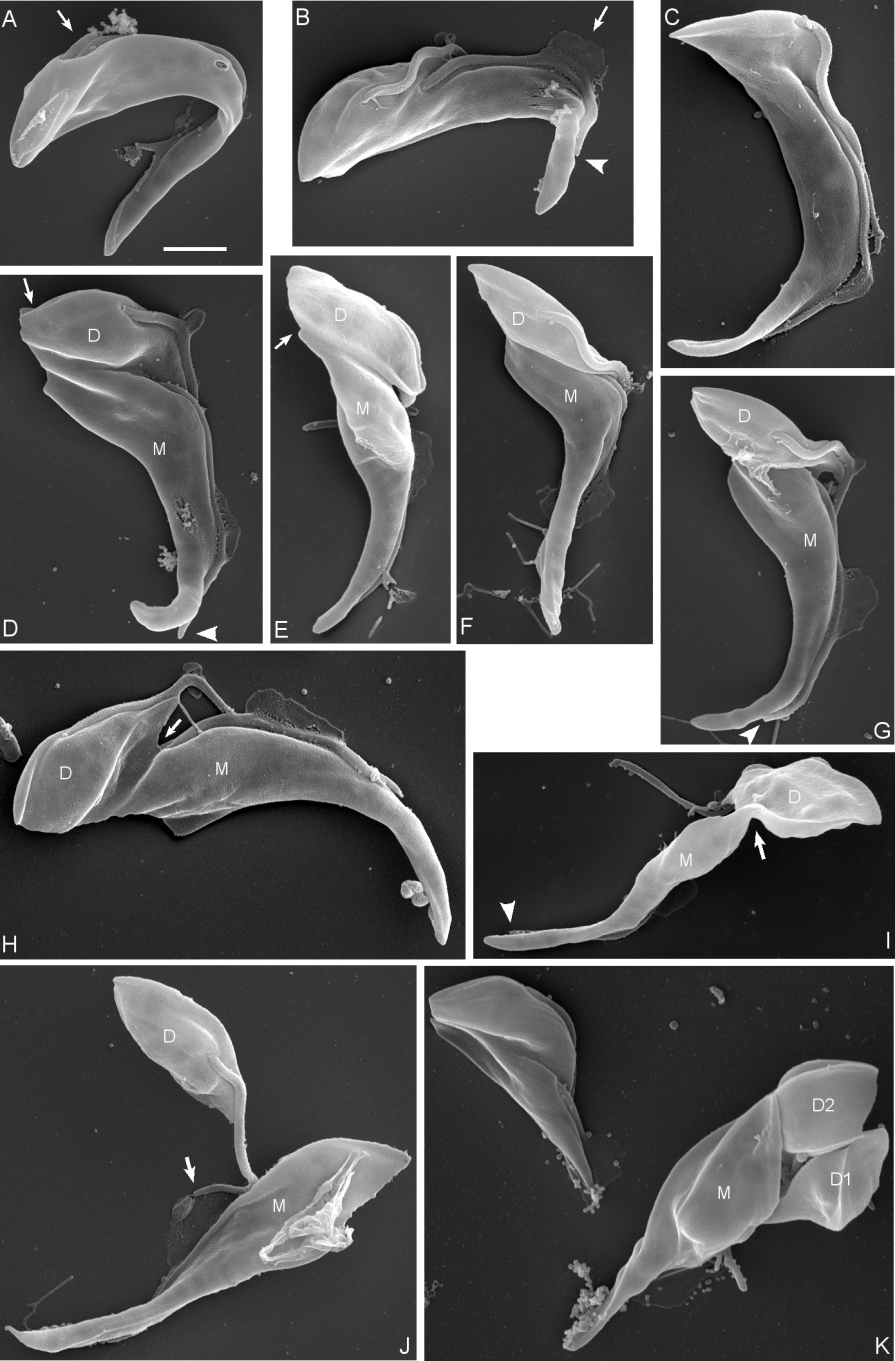
Sequence of events during first division by SEM. Sequence of events during division of proventricular cells of *T*. *congolense* 1/148. **(A)** emergence of the new flagellum (arrow) on the posterior side of the old flagellum. **(B)** separation of the origins of the two flagella. The mid-region of the old flagellum appears to contact the substrate in a pool of matter (arrow). The old flagellum does not extend to the tip of the mother cell body (arrowhead). **(C)** Formation of the cleavage furrow between the two flagella. The origin of the old flagellum sinks into the groove and is no longer visible. **(D)** The cleavage furrow extends further to the posterior and the nascent posterior ends of mother (M) and daughter (D) trypanosomes are visible (arrow). The mid-regions of the M and D flagella are in contact with the substrate in separate pools of matter. The old flagellum does not extend to the tip of the mother cell body (arrowhead). **(E-G)** The shape of the small daughter cell becomes increasingly well-defined during ingression of the cleavage furrow. The nascent posterior ends of mother (M) and daughter (D) trypanosomes are visible in E (arrow). The old flagellum does not extend to the tip of the mother cell body in G (arrowhead). **(H)** Preabscission. The anterior-posterior cleavage furrow is arrowed. **(I)** Preabscission. A broad cytoplasmic bridge remains (arrow). The old flagellum does not extend to the tip of the mother cell body (arrowhead). **(J)** Abscission. The tip of the daughter flagellum appears to be stuck in matter (arrow). **(K)** Mother cell, M, with two sequentially produced daughter cells, D1, D2. To the left is another dividing cell. Scale bar = 2 μm.

To gain a better understanding of this cell division, we studied fixed cells of strain 1/148 YPFR by both light (Fig 8) and scanning electron microscopy (SEM; Fig 9). The first indication of cell replication occurred at T = 14 when elongation and replication of the kinetoplast became evident (Fig 8B). By T = 22, many cells had started to construct a new flagellum, visualized by a dot or short length of YFP::PFR1 in addition to the original flagellum (Fig 8C and 8D). The nucleus then elongated and its division resulted in a 2K2N cell (Fig 8E and 8F). Formation of the cleavage furrow then gave rise to a daughter cell (Fig 8G and 8H). Typically bloodstream form (BSF) and procyclic *T*. *brucei* remain joined by their posterior ends at the final stage of cytokinesis [22], and our observations confirm that this is also the case for *T*. *congolense* savannah BSF and procyclics. However, the posterior-posterior conformation was rarely observed here, and instead the daughter cell remained associated with the mid-region of the mother cell via flagellar contact (Figs 2, 4F, 7 and 8H; S3, S4 and S5 Movies). A second round of division of the mother cell followed rapidly on the first (Fig 8H), resulting in a second daughter cell of similar morphology to the first. All three cells remained adjacent forming a rosette (Fig 8I). The daughter cells were either trypomastigotes (134/424 = 31.6%) or epimastigotes (290/424 = 68.4%) with the kinetoplast close to the nucleus but seldom in the fully anterior position of a canonical epimastigote (Fig 1).

**Fig 10:**
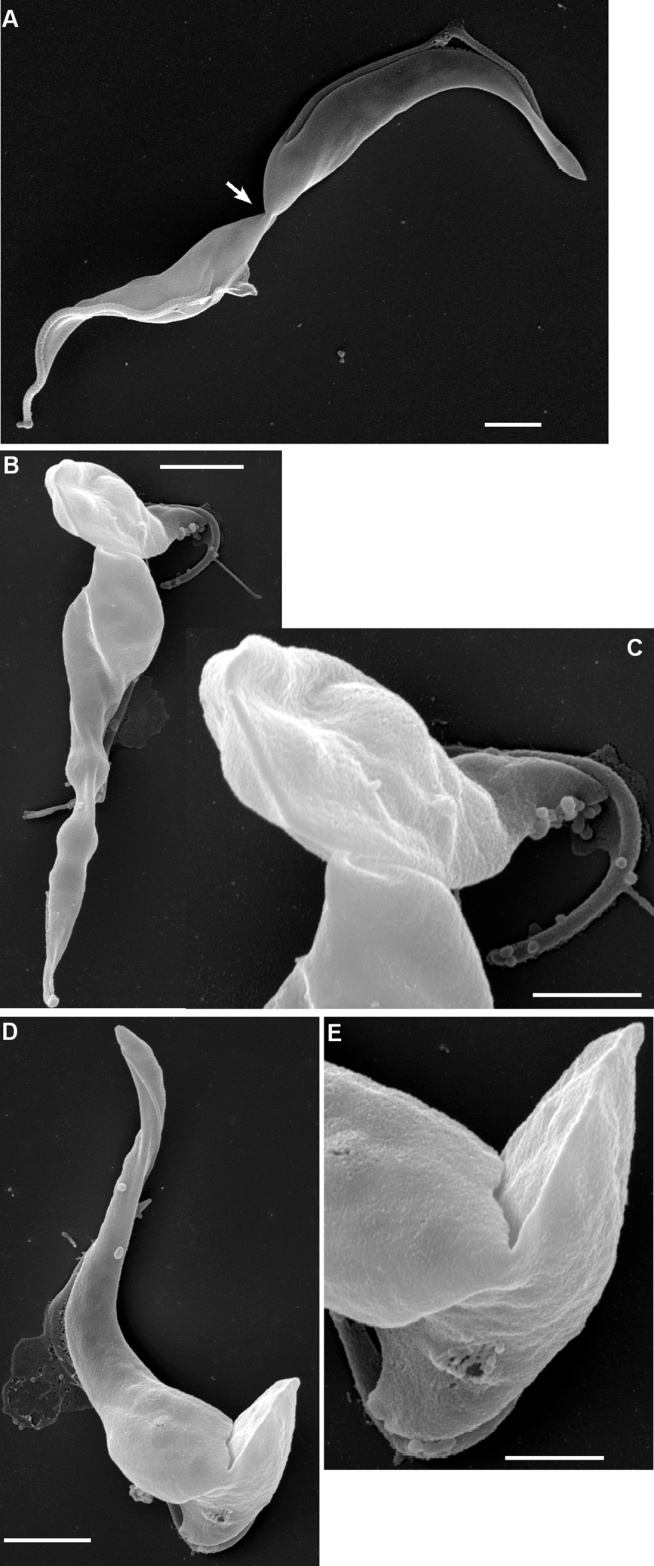
Final stage of abscission. SEM images of proventricular cells of *T*. *congolense* 1/148 during first cell division. **(A)** The posterior-to-posterior conformation with a narrow cytoplasmic bridge connecting the two trypanosomes (arrowed); both cells appear to be trypomastigotes, judging by the exit position of the flagellum, rather than a proventricular cell and daughter; scale bar = 2 μm. **(B)** Proventricular trypanosome with daughter cell (top) joined by a cytoplasmic bridge between the posterior of the mother cell and the mid-region of the daughter; judging by the length of the free flagellum of the daughter, these two cells are about to separate; scale bar = 2 μm. **(C)** Close up of the cytoplasmic bridge; scale bar = 1 μm. **(D)** Proventricular trypanosome with daughter cell (bottom) joined by a cytoplasmic bridge between the posterior of the mother cell and the mid-region of the daughter; scale bar = 2 μm. **(E)** Close up of the cytoplasmic bridge; scale bar = 1 μm.

To investigate cytokinesis in more detail, proventricular cells were examined by SEM. The stage of cytokinesis was interpreted according to the criteria defined for BSF and procyclic *T*. *brucei*: division fold generation, division furrow ingression, preabscission and abscission [22]. The first external evidence of cell division was the emergence of a new flagellum adjacent to the old one, followed by its gradual lengthening and a concomitant widening of the gap between the two flagella (Fig 9A and 9B). In accord with previous descriptions [22], the new flagellum was positioned posterior to the old one and on the left when viewed from the posterior end of the cell. The next externally-visible event was the formation of a cleavage furrow (Fig 9C and 9D). In procyclic and BSF *T*. *brucei*, the cleavage furrow forms between the two flagella, starting at the anterior end of the daughter cell and extending towards the posterior [22]. Likewise, here the furrow appeared just in front of the origin of the old flagellum, defining the small daughter cell. At the same time, some remodelling of the posterior end of the cell occurred, defining the nascent posterior end of the daughter cell (Fig 9D). The daughter cell became increasingly well-defined (Fig 9E–9G) during ingression of the cleavage furrow from the anterior to the posterior. The second stage of cytokinesis is ingression of the cleavage furrow such that a gap appears between the two cells (Fig 9H). In procyclic and BSF *T*. *brucei*, the gap lengthens until only a thin cytoplasmic bridge remains between the two cells at the preabscission stage [22], leading to the characteristic posterior-to-posterior conformation. Here, we found only a single example of this conformation, though both these cells seem to be procyclics (Fig 10A); more commonly, cells were seen with a wide cytoplasmic bridge connecting the posterior of the mother cell with the daughter cell (Figs 9I and 10B) and we postulate that this bridge slides anteriorly on the daughter cell until abscission (Fig 10). It seems likely that the posterior-to-posterior conformation is not possible if the daughter cell remains tethered to the mother cell (Fig 9J).

After abscission was complete, the two cells remained adjacent, both attached to the substrate via their flagella (Fig 9J). A pool of material is evident in the SEM, which is likely to be the zone of attachment in the mid-region of the flagellum of the mother cell. This is also in the same position as the accumulation of PFR1 (S4 Movie), although attachment is not associated with the depot of PFR1 (Fig 5). While the daughter cell usually had a well-shaped, pointed posterior end, the posterior of the mother cell was often misshapen, suggesting little remodelling of the subpellicular microtubules following abscission. This was also evident in some light microscopy images (Fig 7). As the next division cycle of the mother cell begins almost immediately, perhaps there is no time or need for remodelling. The mother cell could be identified with confidence through the first and second cell divisions, but thereafter became difficult to distinguish from other dividing cells in dense clusters of attached cells. The division of daughter cells appeared to be symmetrical (Fig 9K) and similar to that of attached epimastigotes *in vitro*.

## Discussion

### Divergent developmental pathways of *T*. *congolense* and *T*. *brucei*

It has become accepted wisdom that trypanosomes need to undergo an asymmetric division in order to achieve the major morphological rearrangements associated with transition between developmental forms [1,16]. It has even been suggested that the mature flagellum of *T*. *brucei* cannot shorten [16]. Here we show that this conjecture does not hold for the related trypanosome, *T*. *congolense*, during transition from proventricular trypomastigotes to attached epimastigotes, as the flagellum undergoes a 30% reduction in length. In other eukaryotes this would not be regarded as unusual, as flagellar resorption is considered to be a necessary part of the cell cycle [23] and is well documented in the flagellate alga *Chlamydomonas* [24]. The *T*. *congolense* transition from trypomastigote to epimastigote still requires an asymmetric division, but this takes place only after the cell has been remodelled, including shortening of the flagellum. In *T*. *brucei* the equivalent transition is achieved by an asymmetric division resulting in one short and one long epimastigote [3]. While the short epimastigote goes on to colonise the salivary glands, the long epimastigote apparently disintegrates. In contrast, here we found that in *T*. *congolense* the proventricular mother cell undergoes at least two sequential, asymmetric divisions.

**Fig 11:**
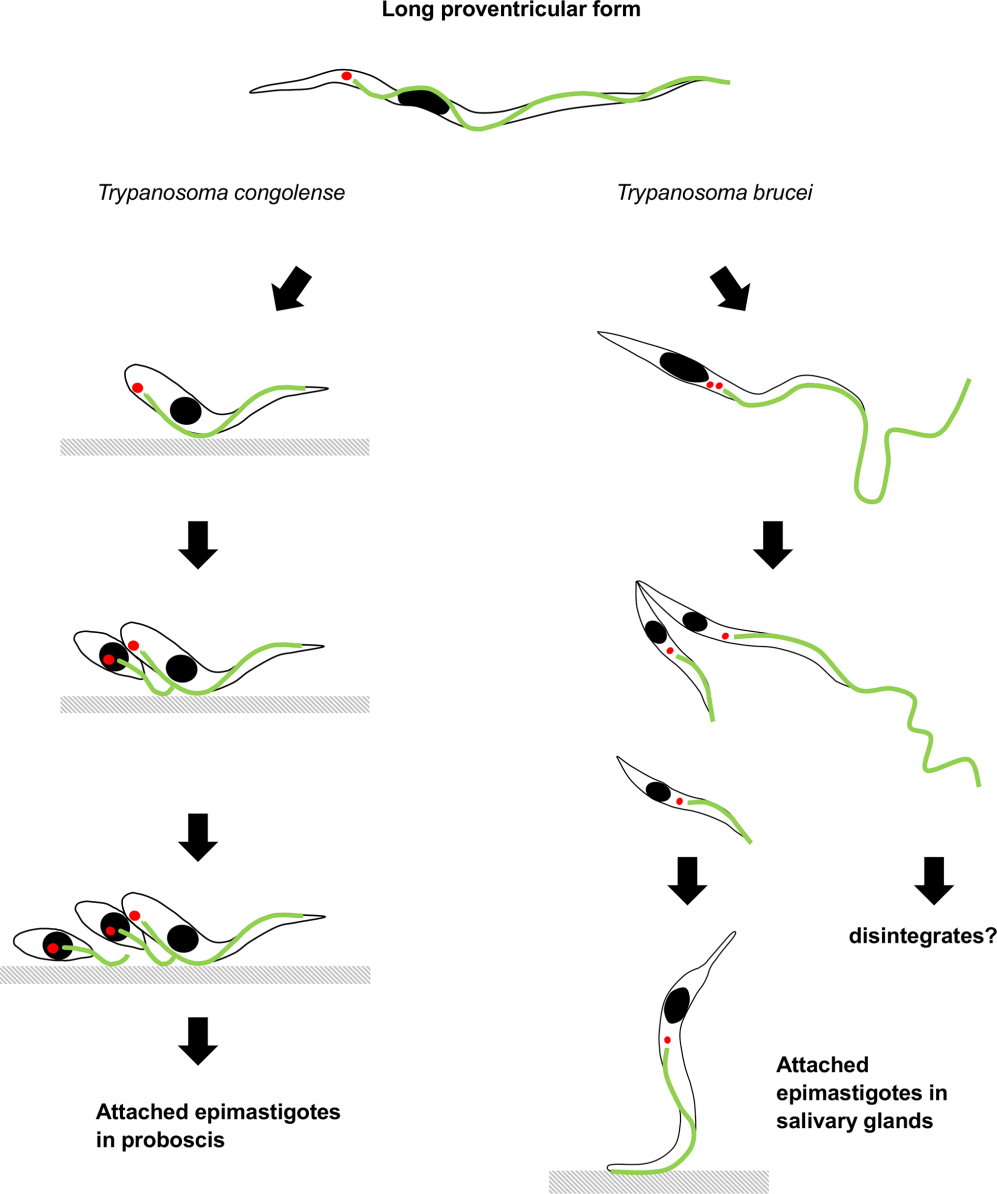
Comparison of the development of proventricular trypanosomes in *Trypanosoma congolense* and *T*. *brucei*. The development of *T*. *congolense* is summarized from the current work, while that for *T*. *brucei* is from [3,38].

Thus these two trypanosome species have evolved quite different approaches to solving the same problem: how to change from a long trypomastigote to become a short epimastigote (Fig 11). Molecular phylogenies group both species in the same clade of African trypanosomes [25,26], but the date of their divergence is unknown. One view of the evolutionary history of the clade is based on sequential stages of adaptation of trypanosomes from the vertebrate bloodstream to transmission by a tsetse vector, with trypanosomes that develop in the salivary glands at the pinnacle; thus, *T*. *brucei* is thought to represent a later stage of evolution than *T*. *congolense* [12], with the expectation that features of development inside the fly will be shared until the pathways diverge. However, this is clearly not the case, as the two species differ fundamentally during the critical but shared step when proventricular trypomastigotes transform to epimastigotes.

### Shortening of the flagellum

Shortening of the mature flagellum, as shown here in proventricular trypomastigotes of *T*. *congolense*, has not been previously reported in trypanosomes [16], and needs a mechanistic explanation. To build the eukaryote flagellum, materials are assembled at the base before transportation to the distal tip by anterograde intraflagellar transport (IFT), and vice versa, deconstruction is accomplished by removal of components from the distal tip and their transport to the base by retrograde IFT [23]. Balance between the opposing IFT systems thus creates a dynamic model for maintenance of flagellar length [27]. However, relatively few flagellar components have been proven to move by IFT [23], and it is by no means certain that this is the sole means of transport.

Trypanosomes have a conventional eukaryote axoneme built of microtubule doublets, which is supported by a flexible rod, the PFR, composed mainly of proteins PFR1 and PFR2 [19], though there are at least 40 proteins associated with the PFR [18]. As both anterograde and retrograde IFT have been demonstrated in trypanosomes [16,28,29], the simplest hypothesis is that the flagellum shortens by increased protein carriage by retrograde IFT, as observed in *Chlamydomonas* [24]. While this satisfactorily explains axoneme deconstruction, our observation that YFP::PFR1 accumulates in the mid-region of the flagellum is not consistent with carriage of PFR1 back to the base of the shortening flagellum by retrograde IFT, unless the IFT trains have been derailed midway. Instead, our results support a model where PFR components are transported independently of IFT. The most compelling evidence for this model comes from the ablation of kinesin 9B expression in *T*. *brucei*, which resulted in production of abnormal flagella with discontinuities in the PFR in daughter cells [30]. In previous experiments it has proved difficult to disentangle the effects of disruption of IFT on axoneme and PFR formation, as these structures are interdependent. In the growing flagellum of *T*. *brucei*, PFR2 is added at the distal tip, but is also incorporated along the length of the flagellum [17]; in addition, there is incorporation of new PFR2 protein into the old flagellum [17]. This may also explain our observation that the intensity of fluorescence of the daughter flagellum of dividing *T*. *brucei* transfected with YFP::PFR1 continues to increase after the flagellum has reached full length [20], though the same phenomenon could not be demonstrated in *T*. *congolense* procyclics, perhaps suggesting that the dynamics of PFR1 addition differs in these two trypanosomes.

### Flagellar attachment

As part of their developmental cycle, epimastigotes of *T*. *congolense* and *T*. *vivax* attach to the interior of the tsetse proboscis and cibarium via the flagellum. In electron micrographs, an electron-dense plaque marks the zone of adhesion between the flagellum and substrate, and a dense network of fibrils fills the space between the flagellar membrane and axoneme [7,31,32]. The same features are evident when *T*. *congolense* epimastigotes attach to a plastic substrate *in vitro* [21,32]. Here *T*. *congolense* trypomastigotes readily attached to glass coverslips *in vitro* after release from the proventriculus. Already, after only an hour of incubation, these attached trypanosomes could not be dislodged by treatment with detergent and calcium chloride, demonstrating the existence of a robust bond like that observed in epimastigotes attached to a plastic substrate [21].

The attachment zone was located in the mid-region of the flagellum, co-incident with the accumulation of a “depot” of PFR1 protein, one of the major building blocks of the PFR. As the depot appeared only after several hours of incubation and was not found in all attached proventricular cells, or their daughters also attached to the substrate, we can conclude that the depot is not essential for attachment, although it may be deposited subsequently at the site of attachment in proventricular trypomastigotes.

### First cell division

After a period of remodelling, each attached proventricular cell produced a daughter trypanosome in an asymmetric division. The daughter was smaller and had a long free flagellum; the kinetoplast was usually in a juxtanuclear position and seldom fully anterior to the nucleus as in the classical epimastigote configuration. As division progressed, the flagellum of the daughter cell formed its own attachment to the substrate. We observed that mother cells quickly re-entered the division cycle and produced a further daughter cell in the same way.

The asymmetric division of proventricular *T*. *congolense* largely followed the canonical pattern described in detail for dividing procyclic and bloodstream form *T*. *brucei* [22]. The cleavage furrow formed between the old and new flagella and ingressed from anterior to posterior, and, despite sometimes observing the separation of the two posterior ends of the mother and daughter cells, we could find no convincing evidence of a cleavage furrow opening in the opposite direction. This alternative route of cytokinesis was described in trypanosomes with ablated expression of cytokinesis initiation factor 1 (CIF1) [33] and the authors suggested that trypanosomes evolved this route as a “backup” in case normal cytokinesis failed. However, it seems more likely to exist for a purpose, considering the extra cost to a microbe where survival depends on the population rather than the individual, and perhaps may yet be found in an obscure life cycle stage where there are constraints on the usual route. Attachment does not appear to pose such a constraint though, as attached epimastigotes of *T*. *brucei* and *T*. *congolense* appear to divide in the normal way with formation of an anterior to posterior cleavage furrow [4,14],

During the preabscission stage, a large cytoplasmic bridge still connected the cell bodies of the mother and daughter cells; this is also seen in *T*. *brucei* BSF and procyclics in preabscission, but the point of contact moves posteriorly until the trypanosomes are joined only by their posterior tips [22]. Here, the last point of contact was between the anterior of the daughter and the posterior of the mother cell, so we postulate that the point of contact slides anteriorly rather than posteriorly on the daughter trypanosome. In unattached trypanosomes such as BSF and procyclics, the biomechanical force required to complete abscission arises from the two trypanosomes swimming in opposite directions. Here, just before abscission, the daughter cell was not yet firmly attached to the substrate, but still joined to the mother cell via the subpellicular microtubules, since their disruption released the daughter cell. At this stage, the daughter cell appears to be tethered to the mother cell but is highly active (S5 Movie) and presumably it is these vigorous movements that finally break the connection between mother and daughter.

## Methods

### Trypanosomes

*T*. *congolense* savannah strains Gam2, WG81, S104 and 1/148, and *T*. *b*. *brucei* J10 were grown as procyclics in Cunningham’s medium (CM) [34] supplemented with 10 μg/ml gentamycin, 5 μg/ml hemin and 15% v/v heat-inactivated foetal calf serum (FCS) at 27°C. Trypanosomes were transfected by electroporation with a plasmid construct designed to tag PFR1 with YFP or cherry RFP at its C terminal end; the construct is integrated into the endogenous PFR1 locus [20]. Transfectants were selected with hygromycin (25 μg/ml) and cloned by limiting dilution.

### Tsetse infection and dissection

Tsetse flies (*Glossina pallidipes*) were kept in single sex groups of 10–20 per cage at 25°C and 70% relative humidity and fed on sterile defibrinated horse blood via a silicone membrane. Male or female flies were given an infected bloodmeal at their first feed 1–5 days after emergence. The infective bloodmeal consisted of procyclic trypanosomes (approximately 10^7^ cells/ml) in CM mixed with an equal volume of washed horse red blood cells resuspended in Hank’s Balanced Salt Solution, supplemented with 10mM L-glutathione to increase infection rates [35]. Flies were cold-anaesthetized before dissection of the gut into a drop of phosphate buffered saline (PBS). The proventriculus was separated from the foregut and midgut using forceps and hypodermic needles and placed in a tube containing CM with 1x anti-contamination cocktail (ACC) [36]. Aliquots of this trypanosome suspension were then added to 1 ml wells of CM with 1x ACC, each well containing a 10–13 mm diameter round glass coverslip. Plates were incubated at 27°C. In initial experiments, we found that trypanosomes developed rosettes of dividing epimastigotes after three or four days but ceased proliferating if left in CM, whereas longevity could be increased by diluting the medium tenfold with PBS containing 20% v/v FCS. Epimastigotes from an established culture are shown in S4 Fig.

### Microscopy

For brightfield microscopy, trypanosomes grown on glass coverslips were washed *in situ* by replacing the medium with PBS; the coverslip was then removed from the well with forceps and allowed to air dry. Cells were fixed with ice cold methanol for 30 s, air dried, mounted in Vectashield (Vector labs) containing 4’,6-diamidino-2-phenylindole (DAPI) and viewed immediately. Images were recorded using a DMRB microscope (Leica) equipped with a Retiga Exi camera and Velocity version 4.1 software (Improvision). Each image was photographed under phase contrast and UV fluorescence at 400x magnification. Measurements were made on the digital images using Image J software (Version 1.41) (*http://rsb.info.nih.gov/ij/*). Dimensions measured are detailed in S1 Fig; measurements of the relative positions of the kinetoplast and nucleus were obtained from DAPI images, while flagellar measurements were obtained from YFP::PFR1 images.

For scanning electron microscopy, trypanosomes grown on glass coverslips were fixed *in situ* by replacing the medium with 2.5% glutaraldehyde in 100mM phosphate buffer pH 7.4, followed by dehydration through an ethanol series and critical-point drying. Coverslips were then placed on a stub and sputter coated with gold/palladium. These samples were viewed on an FEI Quanta 200 field emission SEM.

### Statistical analysis

Morphometric data comprising ten variables measured for each trypanosome from T = 0 to T = 14 hours (S1 Table) were subjected to principal component analysis (PCA) using the *princomp* procedure from the statistical package R (http://www.r-project.org/). Two uncorrelated factors, PC1 and PC2, accounted for most of the variance. Extracted scores for PC1 and PC2 were plotted for each trypanosome from T = 0 to T = 14. Loadings for PC1 and PC2 are given in S4 Table and Fig 6A. In a separate experiment T = 0 to T = 120 minutes (S2 Table), measured variables were scaled and transformed using means, standard deviations and loadings from PCA on T = 0 to T = 14 hours, in order to visualise the data in the same principal component space.

### Preparation of cytoskeletons

Trypanosome cytoskeletons were prepared using 0.5% Triton X-100 to remove the cell membranes, followed by selective depolymerization of the subpellicular microtubules by treatment with 1 mM CaCl_2_, leaving the flagellar axoneme and PFR intact [21].

## Supporting information

S1 Table Morphometrics T = 0 to T = 14 hours. Morphometry of singlet 1K1N *T*. *congolense* cells from pooled proventriculi *in vitro*. The mean ± SE in μm is top line in each box with the range below. Variables as shown in S1 Fig.
(DOCX)

S2 Table Morphometrics T = 0 to T = 120 minutes. Morphometry of singlet 1K1N *T*. *congolense* cells from pooled proventriculi *in vitro*. The mean ± SE in μm is top line in each box with the range below. Variables as shown in S1 Fig.
(DOCX)

S3 Table Morphometrics of flagellum and PFR1 depot T = 0 to T = 14 hours. Measurements of singlet 1K1N *T*. *congolense* cells from pooled proventriculi *in vitro*. The mean ± SE in μm is top line in each box with the range below. Variables: DL, depot length; DPost, distance from depot to cell posterior; DAnt, distance from depot to cell anterior; DAntF, distance from depot to anterior of flagellum (PFR); DPostF, distance from depot to posterior of flagellum (PFR).
(DOCX)

S4 Table Loadings for principal components PC1 and PC2. Variables contributing most to each principal component are in bold.
(DOCX)

S1 Fig Diagram showing measurements made on each trypanosome. The distance from the kinetoplast to the anterior (KAnt) and the distance from the nucleus to the anterior (NAnt) were derived by subtracting KPost or NPost respectively from the length.
(TIF)

S2 Fig Mensural data T = 0 to T = 14 hours. Mean measurements for ten variables plotted against time with standard error bars (see S1 Table).
(TIF)

S3 Fig Comparison of intensity of fluorescence of PFR in flagella of dividing cells. Sequential stages of division in *T*. *brucei* J10 YPFR (A to F) and *T*. *congolense* 1/148 YPFR (G to L). Arrows indicate daughter flagellum. L to R: brightfield, DAPI, YFP, merge. Scale bar = 5 μm.
(TIF)

S4 Fig Analysis of flagellar attachment in cytoskeleton preparations of epimastigotes. A. Fixed epimastigotes of *Trypanosoma congolense* 1/148 YPFR from a culture established from proventricular cells. Cytoskeletons were prepared using 0.5% Triton (row B) or 0.5% Triton follows by CaCl_2_ treatment to selectively remove subpellicular microtubules (row C) [21]. L to R: brightfield, DAPI, merge, YFP, merge. Scale bar = 5 μm.
(TIF)

S5 Fig Mensural data T = 0 to T = 120 minutes. Mean measurements for ten variables plotted against time with standard error bars (see S2 Table).
(TIF)

S1 Movie Attached proventricular trypanosome. Attached cell showing nucleus and kinetoplast stained with Hoechst 33258.
(AVI)

S2 Movie Attachment and remodelling of proventricular cells. Time course from T = 2 to T = 14 hours at ambient temperature (20°C); the lower than normal (27°C) incubation temperature resulted in slight slowing of events. Six proventricular trypanosomes remain attached to the coverslip throughout the time course, while others attach transiently and then move out of the field of view.
(AVI)

S3 Movie Remodelling and first division of attached proventricular cells. Time course from T = 2 to T = 48 at 20°C. Three attached trypanosomes are shown, two of which eventually undergo division to produce a small daughter cell. At the start, the cells are long and attached by their anterior ends; the cells gradually shorten and develop a blunt posterior, which becomes increasingly refractile. The point of attachment shifts from the anterior tip to the mid region of the cell, so that the anterior of the cell becomes free to move again.
(AVI)

S4 Movie PFR1 depot in live cells. Trypanosomes (1/148 YFP) from the proventriculus undergoing first asymmetric division. The first part of the movie shows trypanosomes imaged by phase contrast microscopy, followed by visualisation of YFP::PFR1 by fluorescence. Accumulation of YFP::PFR1 is evident in the mother cells only and co-localizes with the region of attachment of the mother flagellum to the glass coverslip.
(AVI)

S5 Movie Asymmetric division *in vitro*. Trypanosomes from the proventriculus. The cell on the right has already produced a daughter cell.
(AVI)

S6 Movie Asymmetric division *in vivo*. Trypanosomes from a tsetse proboscis; the morphology of this dividing cell is identical to that seen for proventricular cells *in vitro*.
(AVI)

S1 File Principal component analysis. Sequential timepoints of the Principal Component Analysis for individual trypanosome cells from T = 0 to T = 14. Each circle represents one cell; black filled circles are specific to the timepoint shown. Animated GIF.
(GIF)
