# Supplementary material for: Shape-shifting trypanosomes: Flagellar shortening followed by asymmetric division in Trypanosoma congolense from the tsetse proventriculus
Source: PLoS Pathog. 2018 May 17;14(5):e1007043. doi: 10.1371/journal.ppat.1007043 (PMC5957336; doi:10.1371/journal.ppat.1007043)
Supplement: S1 Table — Morphometry of singlet 1K1N T. congolense cells from pooled proventriculi in vitro. The mean ± SE in μm is top line in each box with the range below. Variables as shown in S1 Fig. (DOCX) [file ppat.1007043.s001.docx]

**S1 Table. Morphometrics T=0 to T=14 hours.** Morphometry of singlet 1K1N *T. congolense* cells from pooled proventriculi *in vitro*. The mean ± SE in µm is top line in each box with the range below. Variables as shown in S1 Fig.

| Time (No.) | Length | Width | KPost | KNuc | NPost | NL | NW | KAnt | NAnt | FL |
| --- | --- | --- | --- | --- | --- | --- | --- | --- | --- | --- |
| 0  (101) | 32.32 ± 0.39  21.48-41.78 | 1.50 ± 0.02  1.03-2.28 | 4.20 ± 0.16  0.46-8.28 | 3.08 ± 0.07  1.45-4.75 | 7.94 ± 0.17  3.74-12.68 | 3.27 ± 0.05  1.82-4.68 | 1.20 ± 0.02  0.80-1.90 | 28.12 ± 0.31  18.84-33.79 | 24.38 ± 0.32  14.27-30.83 | 26.59 ± 0.33  16.32-32.70 |
| 2  (114) | 32.33 ± 0.34  24.03-45.80 | 1.87 ± 0.03  1.31-2.82 | 1.35 ± 0.11  0.00-5.39 | 2.82 ± 0.06  0.99-4.54 | 4.98 ± 0.13  1.96-9.92 | 4.18 ± 0.06  2.65-6.14 | 1.53 ± 0.02  1.00-2.43 | 30.98 ± 0.30  23.95-40.70 | 27.34 ± 0.31  20.49-36.28 | 30.29 ± 0.34  20.06-40.99 |
| 4  (130) | 29.88 ± 0.30  23.49-42.00 | 2.08 ± 0.03  1.34-3.59 | 0.55 ± 0.06  0.00-3.79 | 2.68 ± 0.06  0.51-4.98 | 4.02 ± 0.10  1.36-8.28 | 3.88 ± 0.05  2.61-5.67 | 1.64 ± 0.02  0.98-2.54 | 29.33 ± 0.29  22.61-39.81 | 25.86 ± 0.27  20.14-35.70 | 29.36 ± 0.32  20.54-40.86 |
| 6  (94) | 27.06 ± 0.41  19.42-41.52 | 2.40 ± 0.04  1.60-3.79 | 0.29 ± 0.05  0.00-3.05 | 2.50 ± 0.07  0.67-4.19 | 3.63 ± 0.09  1.54-6.10 | 3.51 ± 0.05  1.92-5.12 | 1.86 ± 0.03  1.21-2.72 | 26.77 ± 0.40  19.42-40.57 | 23.43 ± 0.38  15.60-35.71 | 26.80 ± 0.42  17.35-41.64 |
| 10  (103) | 23.19 ± 0.38  16.90-38.55 | 2.48 ± 0.04  1.59-3.60 | 0.42 ± 0.05  0.00-2.13 | 2.48 ± 0.06  0.59-4.37 | 3.74 ± 0.07  1.75-5.34 | 3.36 ± 0.06  2.24-6.07 | 1.86 ± 0.03  1.27-2.84 | 22.76 ± 0.37  15.68-36.42 | 19.44 ± 0.36  11.77-33.23 | 21.79 ± 0.41  14.98-36.07 |
| 12  (73) | 22.62 ± 0.38  16.11-30.21 | 2.82 ± 0.05  1.61-3.91 | 0.60 ± 0.07  0.00-2.84 | 2.49 ± 0.05  1.34-3.41 | 3.93 ± 0.08  2.61-6.06 | 3.33 ± 0.06  2.57-5.26 | 2.06 ± 0.04  1.31-2.98 | 22.01 ± 0.39  15.09-30.21 | 18.69 ± 0.38  12.45-26.45 | 20.50 ± 0.44  13.38-29.76 |
| 14  (89) | 22.36 ± 0.46  16.04-35.66 | 2.73 ± 0.05  1.73-4.00 | 1.07 ± 0.07  0.00-2.84 | 2.26 ± 0.06  0.85-3.49 | 4.15 ± 0.09  1.85-6.25 | 3.36 ± 0.06  2.30-4.73 | 1.93 ± 0.04  1.08-2.66 | 21.29 ± 0.45  14.30-33.78 | 18.21 ± 0.44  11.38-30.50 | 20.11 ± 0.49  12.38-33.27 |
