# Supplementary material for: Shape-shifting trypanosomes: Flagellar shortening followed by asymmetric division in Trypanosoma congolense from the tsetse proventriculus
Source: PLoS Pathog. 2018 May 17;14(5):e1007043. doi: 10.1371/journal.ppat.1007043 (PMC5957336; doi:10.1371/journal.ppat.1007043)
Supplement: S2 Table — Morphometry of singlet 1K1N T. congolense cells from pooled proventriculi in vitro. The mean ± SE in μm is top line in each box with the range below. Variables as shown in S1 Fig. (DOCX) [file ppat.1007043.s002.docx]

**S2 Table. Morphometrics T=0 to T=120 minutes.** Morphometry of singlet 1K1N *T. congolense* cells from pooled proventriculi *in vitro*. The mean ± SE in µm is top line in each box with the range below. Variables as shown in S1 Fig.

| Time (mins) (No.) | L | W | KPost | KNuc | NPost | NL | NW | KAnt | NAnt | FL |
| --- | --- | --- | --- | --- | --- | --- | --- | --- | --- | --- |
| 0  (100) | 33.70 ± 0.35  24.81-40.28 | 1.79 ± 0.03  1.10-2.69 | 4.28 ± 0.12  1.30-8.28 | 3.11 ± 0.06  1.85-5.11 | 8.27 ± 0.14  4.82-11.91 | 3.68 ± 0.05  2.51-4.89 | 1.58 ± 0.03  1.05-2.44 | 29.42 ± 0.29  22.24-36.06 | 25.43 ± 0.30  18.26-31.71 | 28.38 ± 0.32  20.84-35.47 |
| 30  (28) | 37.68 ± 0.62  31.77-43.28 | 1.78 ± 0.04  1.42-2.28 | 3.58 ± 0.26  0.96-7.02 | 3.44 ± 0.15  2.13-4.75 | 7.89 ± 0.31  4.90-11.07 | 3.99 ± 0.10  3.08-5.27 | 1.47 ± 0.03  1.10-1.82 | 34.09 ± 0.61  29.36-40.59 | 29.78 ± 0.60  24.57-37.35 | 32.95 ± 0.59  28.14-38.61 |
| 60  (63) | 37.30 ± 0.56  27.53-48.06 | 1.92 ± 0.03  1.41-2.80 | 2.82 ± 0.16  0.00-6.63 | 3.34 ± 0.10  1.31-5.10 | 7.27 ± 0.19  4.17-12.29 | 4.19 ± 0.07  3.15-5.70 | 1.68 ± 0.04  0.96-2.51 | 34.48 ± 0.48  24.70-43.20 | 30.03 ± 0.49  20.69-38.24 | 33.51 ± 0.52  23.85-41.68 |
| 90  (71) | 35.26 ± 0.44  25.69-44.64 | 1.94 ± 0.03  1.36-2.67 | 1.86 ± 0.17  0.00-6.53 | 3.25 ± 0.09  1.03-6.28 | 6.11 ± 0.20  2.41-11.46 | 4.34 ± 0.06  2.88-5.37 | 1.75 ± 0.03  1.27-2.53 | 33.40 ± 0.44  23.47-42.18 | 29.15 ± 0.41  19.15-37.22 | 32.76 ± 0.46  23.21-41.97 |
| 120  (56) | 34.40 ± 0.48  25.45-48.41 | 2.06 ± 0.05  1.26-3.36 | 1.24 ± 0.17  0.00-6.47 | 2.90 ± 0.04  1.57-5.47 | 5.18 ± 0.11  3.20-12.28 | 4.48 ± 0.12  3.03-9.08 | 1.83 ± 0.04  1.14-2.76 | 33.16 ± 0.43  23.80-41.94 | 29.22 ± 0.44  20.18-36.13 | 32.90 ± 0.47  23.76-41.42 |
