# Supplementary material for: Shape-shifting trypanosomes: Flagellar shortening followed by asymmetric division in Trypanosoma congolense from the tsetse proventriculus
Source: PLoS Pathog. 2018 May 17;14(5):e1007043. doi: 10.1371/journal.ppat.1007043 (PMC5957336; doi:10.1371/journal.ppat.1007043)
Supplement: S3 Table — Measurements of singlet 1K1N T. congolense cells from pooled proventriculi in vitro. The mean ± SE in μm is top line in each box with the range below. Variables: DL, depot length; DPost, distance from depot to cell posterior; DAnt, distance from depot to cell anterior; DAntF, distance from depot to anterior of flagellum (PFR); DPostF, distance from depot to posterior of flagellum (PFR). (DOCX) [file ppat.1007043.s003.docx]

**S3 Table. Morphometrics of flagellum and depot T=0 to T=14 hours.** Measurements of singlet 1K1N *T. congolense* cells from pooled proventriculi *in vitro*. The mean ± SE in µm is top line in each box with the range below. Variables: DL, depot length; DPost, distance from depot to cell posterior; DAnt, distance from depot to cell anterior; DAntF, distance from depot to anterior of flagellum (PFR); DPostF, distance from depot to posterior of flagellum (PFR).

| Time (No.) | DL | DPost | DAnt | DAntF | DPostF |
| --- | --- | --- | --- | --- | --- |
| 0  (0) | 0.00 ± 0.00  0.00 -0.00 | 0.00 ± 0.00  0.00 -0.00 | 0.00 ± 0.00  0.00 -0.00 | 0.00 ± 0.00  0.00 -0.00 | 0.00 ± 0.00  0.00 -0.00 |
| 2  (0) | 0.00 ± 0.00  0.00 -0.00 | 0.00 ± 0.00  0.00 -0.00 | 0.00 ± 0.00  0.00 -0.00 | 0.00 ± 0.00  0.00 -0.00 | 0.00 ± 0.00  0.00 -0.00 |
| 4  (20) | 0.99 ± 0.07  0.57-1.92 | 18.90 ± 0.37  16.38-22.32 | 10.08 ± 0.50  5.90-13.38 | 10.39 ± 0.62  5.90-17.11 | 17.86 ± 0.72  9.50-22.67 |
| 6  (22) | 1.48 ± 0.12  0.54-3.01 | 14.68 ± 0.49  9.92-19.22 | 10.42 ± 0.49  4.56-14.50 | 10.29 ± 0.49  5.58-14.30 | 14.34 ± 0.60  8.38-19.86 |
| 10  (50) | 1.58 ± 0.05  0.81-2.34 | 11.46 ± 0.34  2.64-18.95 | 10.03 ± 0.34  6.30-16.40 | 8.69 ± 0.40  0.24-15.44 | 10.87 ± 0.37  7.11-20.12 |
| 12  (47) | 1.80 ± 0.06  0.98-2.79 | 10.64 ± 0.52  0.00-21.01 | 10.68 ± 0.52  0.49-20.50 | 9.83 ± 0.62  4.17-25.02 | 8.86 ± 0.50  0.81-14.96 |
| 14  (55) | 1.64 ± 0.05  0.91-2.54 | 10.21 ± 0.64  0.36-29.63 | 9.73 ± 0.58  0.00-26.27 | 8.40 ± 0.62  0.00-27.27 | 8.88 ± 0.60  0.75-27.58 |
