# Supplementary material for: Shape-shifting trypanosomes: Flagellar shortening followed by asymmetric division in Trypanosoma congolense from the tsetse proventriculus
Source: PLoS Pathog. 2018 May 17;14(5):e1007043. doi: 10.1371/journal.ppat.1007043 (PMC5957336; doi:10.1371/journal.ppat.1007043)
Supplement: S4 Table — Variables contributing most to each principal component are in bold. (DOCX) [file ppat.1007043.s004.docx]

**S4 Table. Loadings for principal components PC1 and PC2.** Variables contributing most to each principal component are in bold.

| **Variable** | **PC1** | **PC2** |
| --- | --- | --- |
| KAnt | **-0.413** | -0.226 |
| NAnt | **-0.402** | -0.268 |
| KNuc | -0.194 | 0.302 |
| log KPost | -0.172 | **0.461** |
| Length | **-0.433** | -0.077 |
| FL | **-0.394** | -0.264 |
| log NL | -0.176 | -0.351 |
| Width | 0.312 | -0.194 |
| NW | 0.261 | -0.306 |
| log NPost | -0.248 | **0.493** |
