# Supplementary figures and images for: Shape-shifting trypanosomes: Flagellar shortening followed by asymmetric division in Trypanosoma congolense from the tsetse proventriculus
Source: PLoS Pathog. 2018 May 17;14(5):e1007043. doi: 10.1371/journal.ppat.1007043 (PMC5957336; doi:10.1371/journal.ppat.1007043)

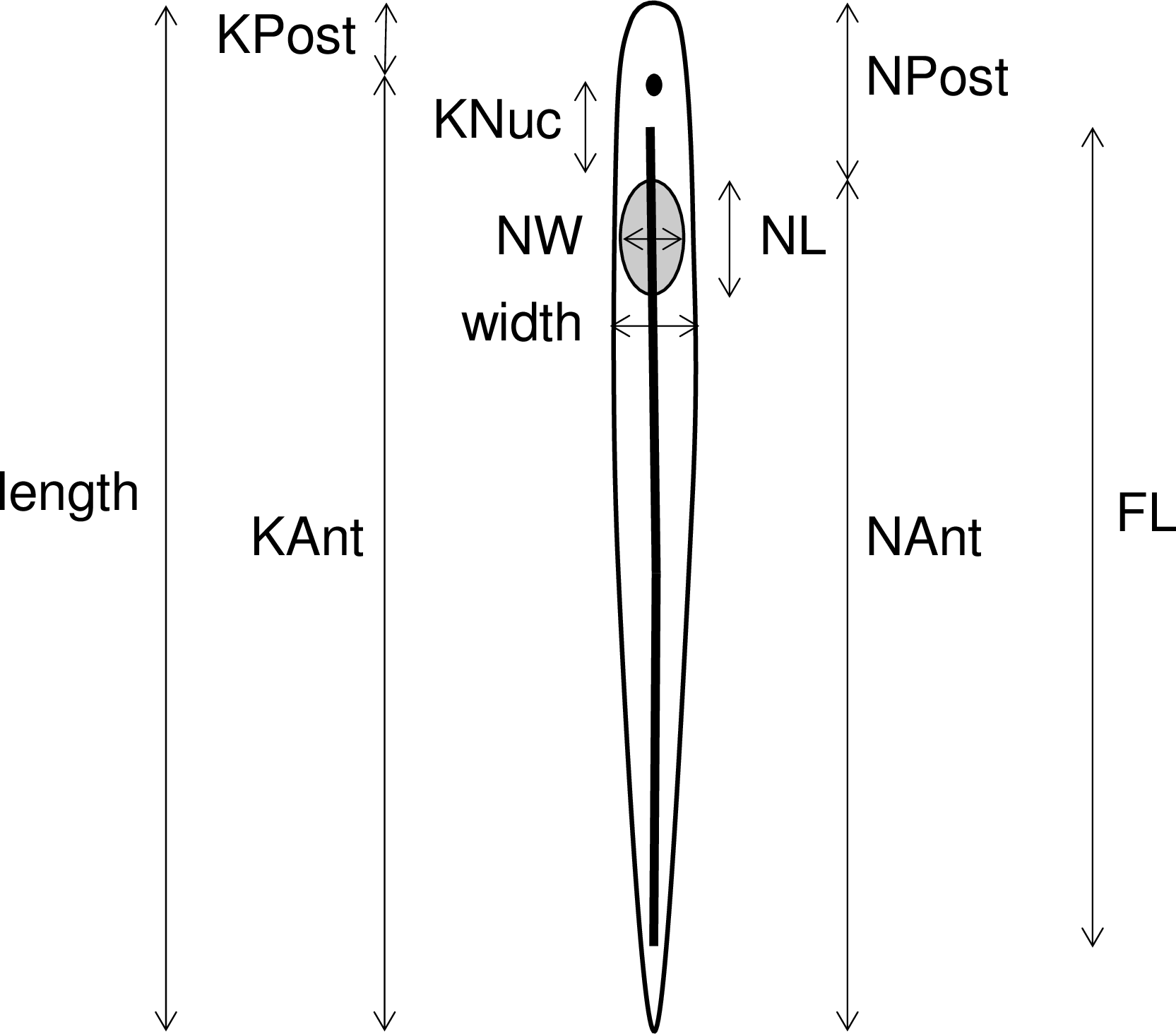

Supplement: S1 Fig — The distance from the kinetoplast to the anterior (KAnt) and the distance from the nucleus to the anterior (NAnt) were derived by subtracting KPost or NPost respectively from the length. (TIF) [file ppat.1007043.s005.tif]

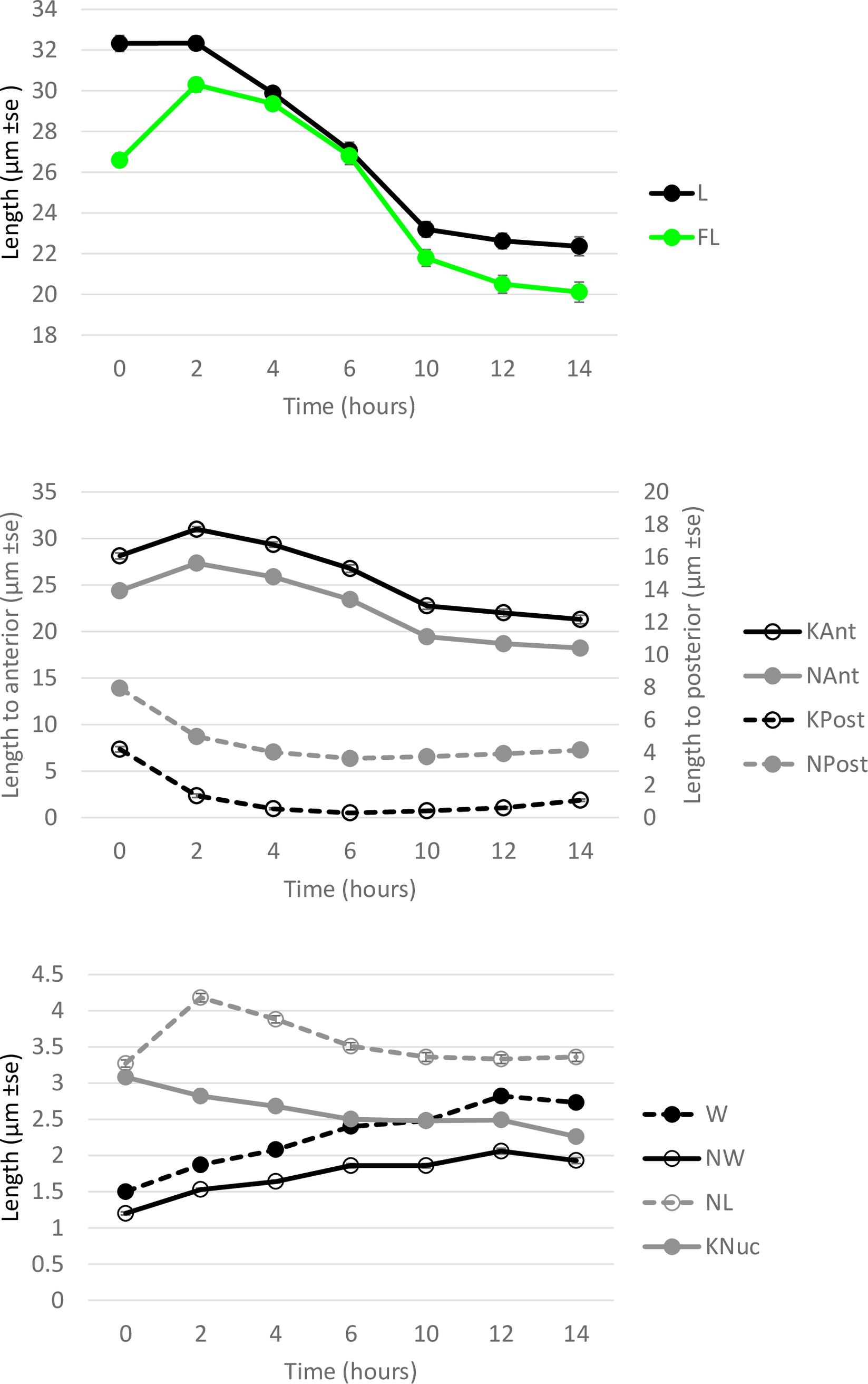

Supplement: S2 Fig — Mean measurements for ten variables plotted against time with standard error bars (see S1 Table). (TIF) [file ppat.1007043.s006.tif]

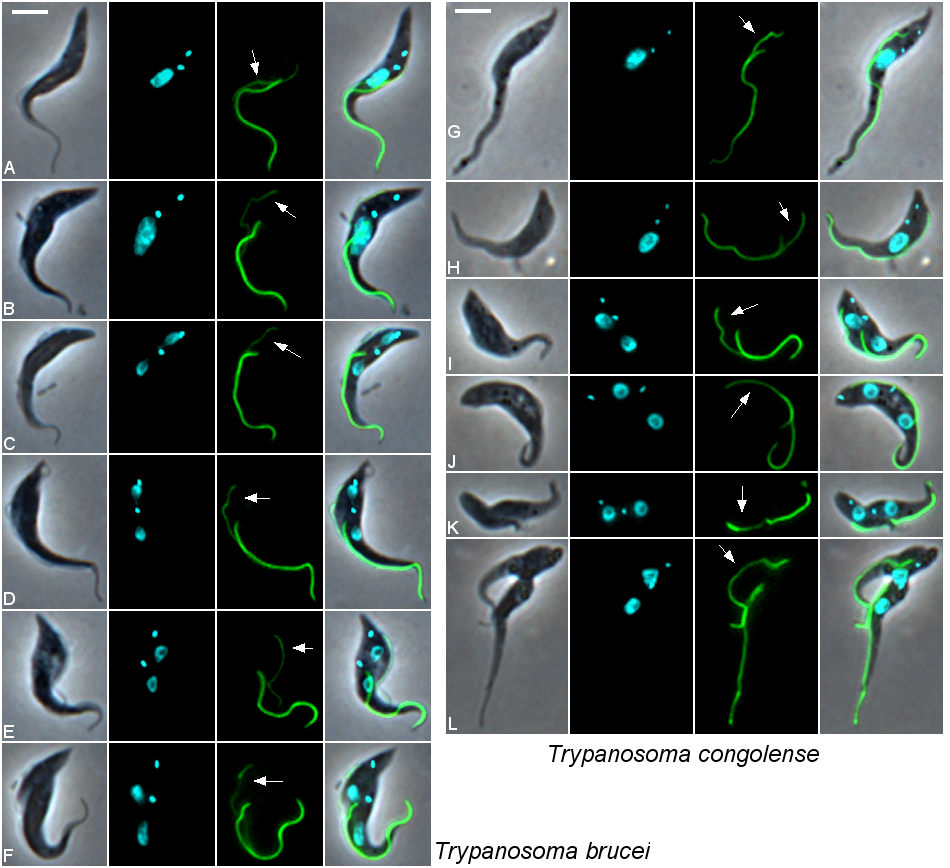

Supplement: S3 Fig — Sequential stages of division in T. brucei J10 YPFR (A to F) and T. congolense 1/148 YPFR (G to L). Arrows indicate daughter flagellum. L to R: brightfield, DAPI, YFP, merge. Scale bar = 5 μm. (TIF) [file ppat.1007043.s007.tif]

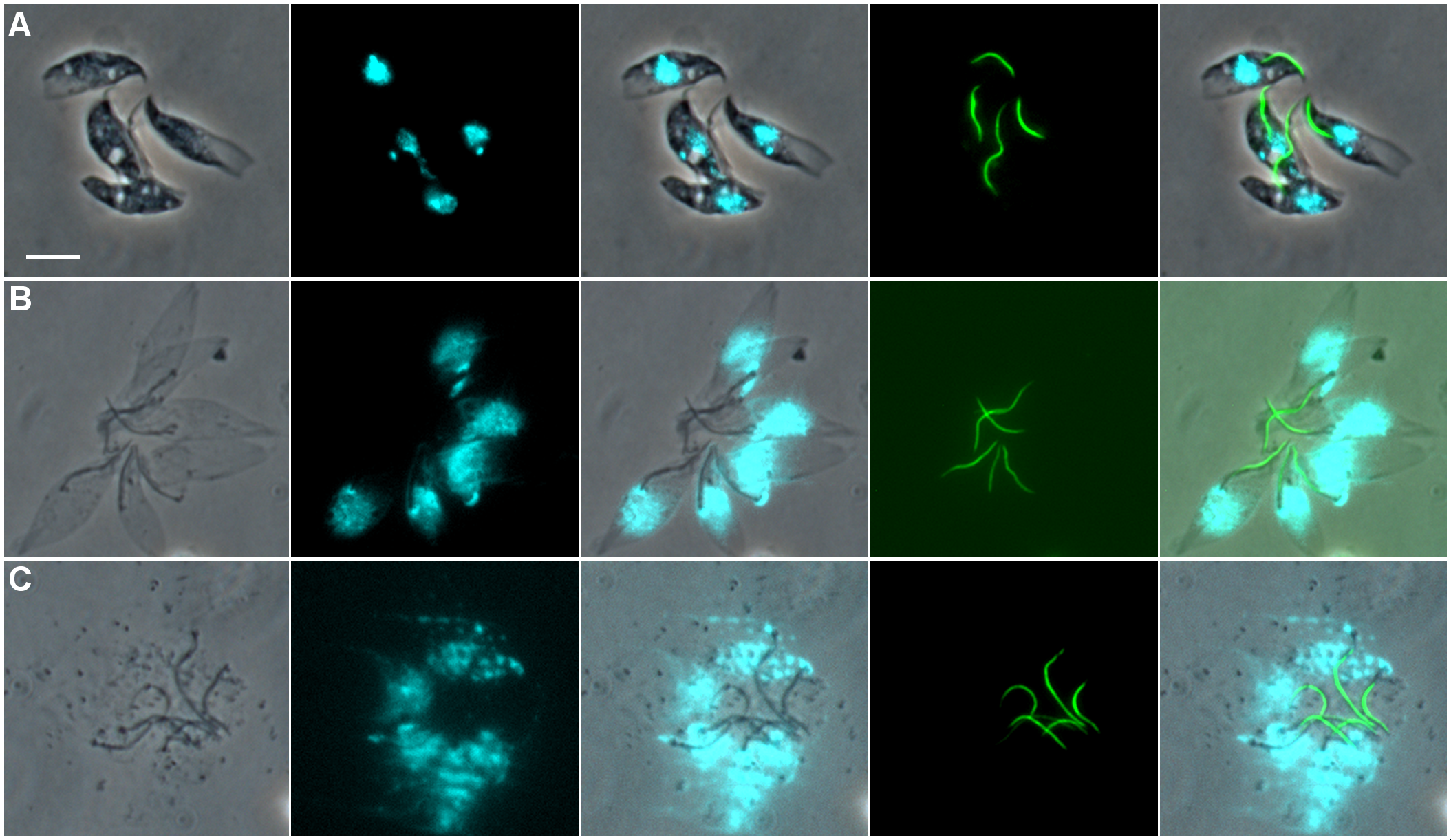

Supplement: S4 Fig — A. Fixed epimastigotes of Trypanosoma congolense 1/148 YPFR from a culture established from proventricular cells. Cytoskeletons were prepared using 0.5% Triton (row B) or 0.5% Triton follows by CaCl2 treatment to selectively remove subpellicular microtubules (row C) [21]. L to R: brightfield, DAPI, merge, YFP, merge. Scale bar = 5 μm. (TIF) [file ppat.1007043.s008.tif]

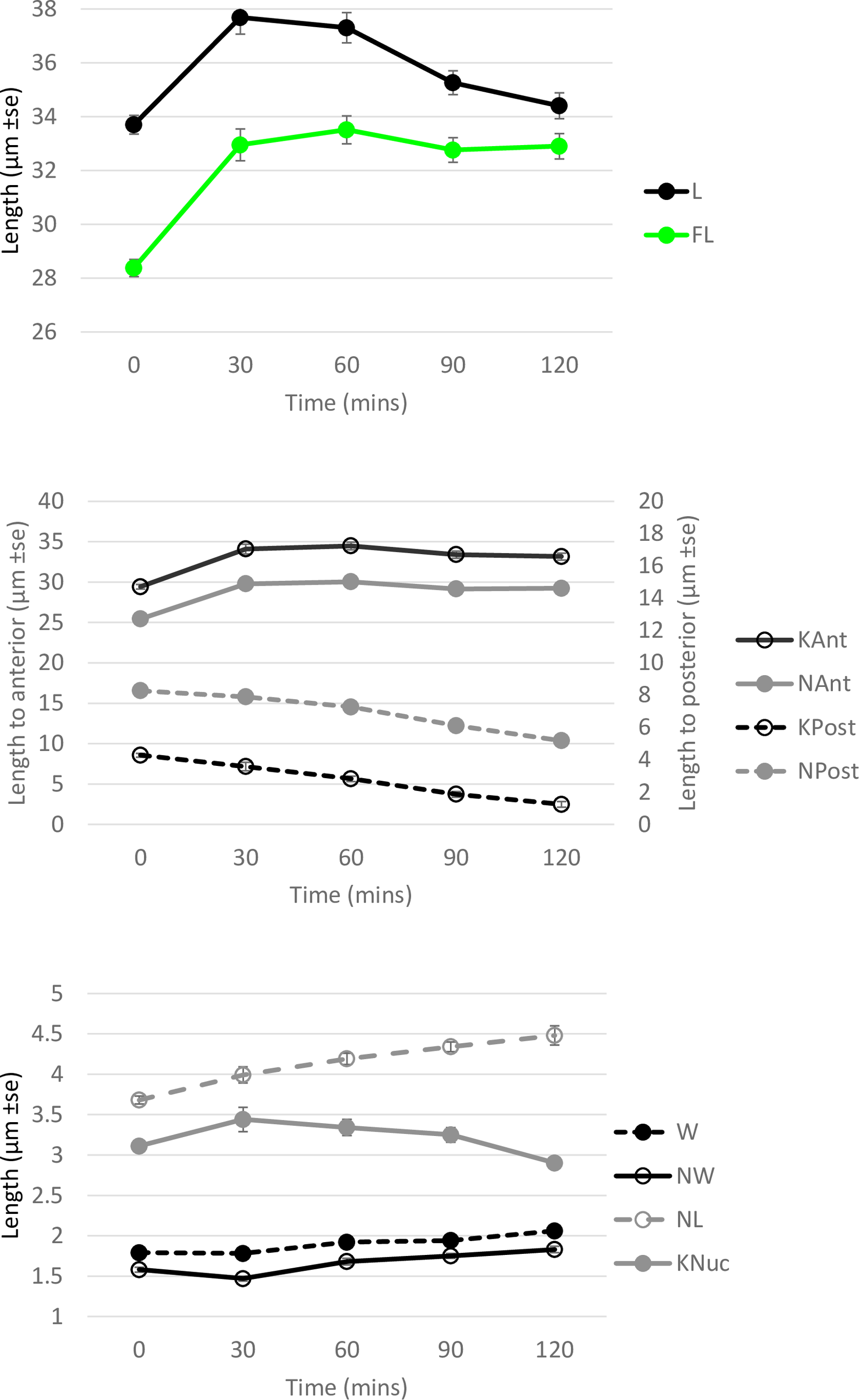

Supplement: S5 Fig — Mean measurements for ten variables plotted against time with standard error bars (see S2 Table). (TIF) [file ppat.1007043.s009.tif]

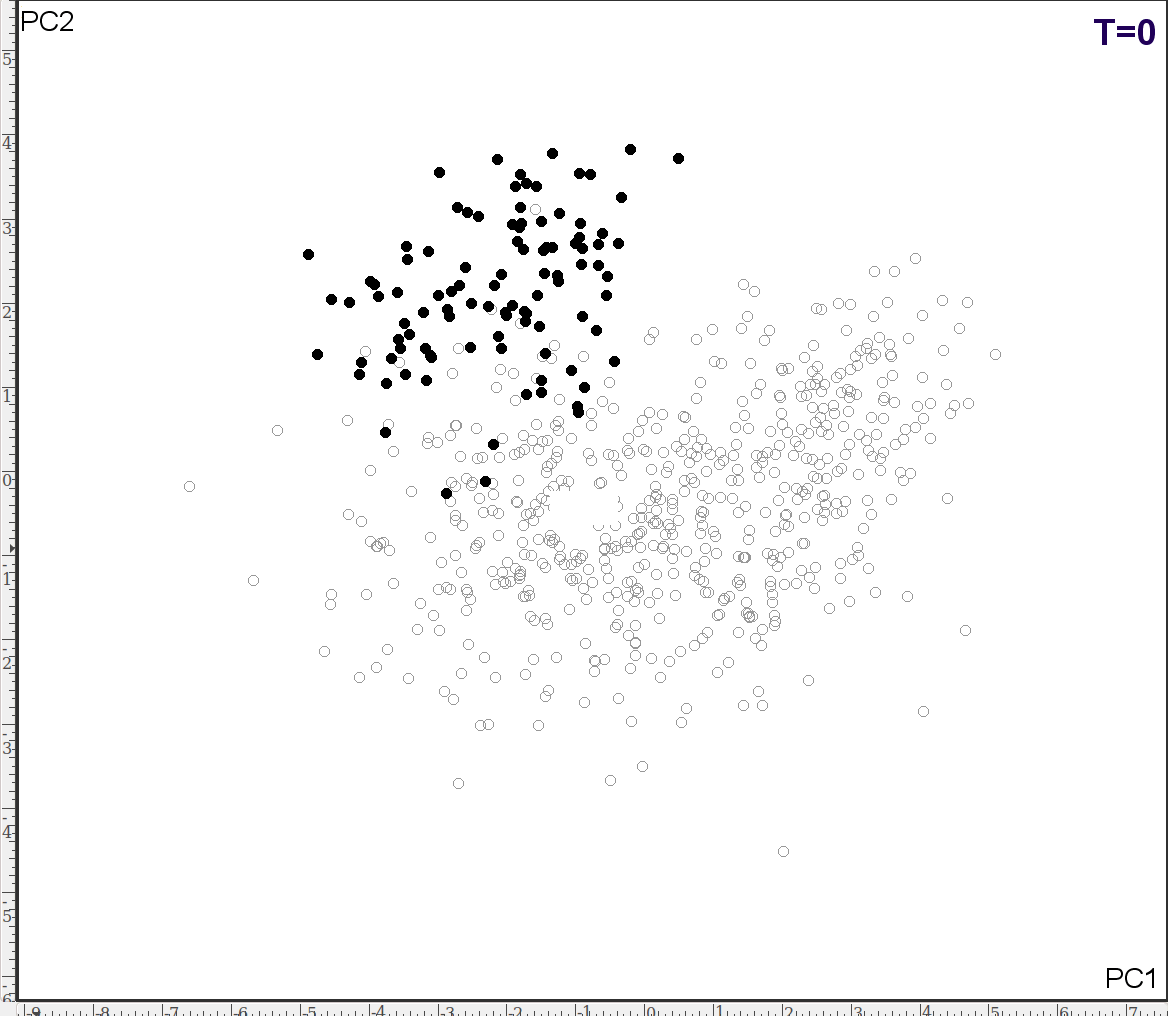

Supplement: S1 File — Sequential timepoints of the Principal Component Analysis for individual trypanosome cells from T = 0 to T = 14. Each circle represents one cell; black filled circles are specific to the timepoint shown. Animated GIF. (GIF) [file ppat.1007043.s016.gif]
